# A scoping review of smoking cessation pharmacogenetic studies to advance future research across racial, ethnic, and ancestral populations

**DOI:** 10.3389/fgene.2023.1103966

**Published:** 2023-06-08

**Authors:** Elizabeth C. Prom-Wormley, Jonathan L. Wells, Lori Landes, Amy N. Edmondson, Mariam Sankoh, Brendan Jamieson, Kayla J. Delk, Sanya Surya, Shambhavi Bhati, James Clifford

**Affiliations:** ^1^ Division of Epidemiology, Department of Family Medicine and Population Health, Virginia Commonwealth University, Richmond, VA, United States; ^2^ Department of Family Medicine and Population Health, Virginia Commonwealth University, Richmond, VA, United States; ^3^ Department of Integrative Life Sciences, Virginia Commonwealth University, Richmond, VA, United States; ^4^ Department of Public Health, Brody School of Medicine, East Carolina University, Greenville, United States

**Keywords:** smoking cessation, pharmacotherapy, genetics, racial, ethnic, cigarettes, pharmacogenomic

## Abstract

Abstinence rates among smokers attempting to quit remain low despite the wide availability and accessibility of pharmacological smoking cessation treatments. In addition, the prevalence of cessation attempts and abstinence differs by individual-level social factors such as race and ethnicity. Clinical treatment of nicotine dependence also continues to be challenged by individual-level variability in effectiveness to promote abstinence. The use of tailored smoking cessation strategies that incorporate information on individual-level social and genetic factors hold promise, although additional pharmacogenomic knowledge is still needed. In particular, genetic variants associated with pharmacological responses to smoking cessation treatment have generally been conducted in populations with participants that self-identify as White race or who are determined to be of European genetic ancestry. These results may not adequately capture the variability across all smokers as a result of understudied differences in allele frequencies across genetic ancestry populations. This suggests that much of the current pharmacogenetic study results for smoking cessation may not apply to all populations. Therefore, clinical application of pharmacogenetic results may exacerbate health inequities by racial and ethnic groups. This scoping review examines the extent to which racial, ethnic, and ancestral groups that experience differences in smoking rates and smoking cessation are represented in the existing body of published pharmacogenetic studies of smoking cessation. We will summarize results by race, ethnicity, and ancestry across pharmacological treatments and study designs. We will also explore current opportunities and challenges in conducting pharmacogenomic research on smoking cessation that encourages greater participant diversity, including practical barriers to clinical utilization of pharmacological smoking cessation treatment and clinical implementation of pharmacogenetic knowledge.

## Introduction

Smoking remains a leading factor of preventable death and disease. Approximately 16 million Americans have a smoking-related illness, leading to over 480,000 deaths annually (United States Public Health Service Office of the Surgeon General, National Center for Chronic Disease Prevention and Health Promotion US Office on Smoking and Health, 2020). Consequently, cigarette smoking costs the United States more than $600 billion each year, including over $240 billion in healthcare spending (Shrestha et al., 2022). Increasing the number of smokers who successfully make quit attempts and remain abstinent for a year or longer through the use of pharmacological treatment is therefore a common strategy to reduce the burden of smoking on society.

Only 6–7% of adult smokers who make a quit attempt are successful each year (Creamer et al., 2019). Consequently, improving population-level smoking quit attempt success to reduce the burden of smoking is a public health goal that remains elusive but may be addressed through development of personalized cessation strategies that take specific genetic and social environmental factors influencing smoking behaviors and cessation outcomes into account.

Prior studies consistently report that the genetic and environmental factors that influence smoking cessation are also shared with other smoking behaviors (Richmond-Rakerd et al., 2016). Evidence from twin studies report the role of genetic and environmental influences on various smoking behaviors (Rose, 2009) including smoking initiation (Li et al., 2003), age of initiation (Maes et al., 2017), the quantity of cigarettes smoked (Li et al., 2003; Richmond-Rakerd et al., 2014), nicotine dependence (Maes et al., 2011), and cessation (Xian et al., 2003). Genetic association studies have identified several genetic factors associated with smoking behaviors (Furberg et al., 2010; Liu et al., 2019; Quach et al., 2020; Xu et al., 2021) and cessation (Liu et al., 2019; Erzurumluoglu et al., 2020; Xu et al., 2020) measured as being a current *versus* former smoker. Specific environmental factors have also been associated with smoking behaviors (e.g., having peers who use tobacco (Harden et al., 2008), household income (McMillen et al., 2015), and exposure to tobacco marketing (Choi et al., 2020; Lee et al., 2015)). Additionally, several environmental factors have been associated with cessation and abstinence (De Viron et al., 2013). Genetic association studies have reported several variants associated with cessation (e.g., former vs. current smoker) that have also been associated with smoking behaviors (Liu et al., 2019).

Although smoking cessation treatments were developed to support all smokers, differences in cessation rates exist across age (Solberg et al., 2007; Jamal et al., 2015; 2018; Babb et al., 2017; Creamer et al., 2019), sex (Li, 2003; Madden et al., 2004; Piper et al., 2010; Smith et al., 2016), education (Pennanen et al., 2014; Lund, 2015; Babb et al., 2017; Jamal et al., 2018; Ruokolainen et al., 2021), marital status (Ramsey et al., 2019; Pennanen et al., 2014; Broms et al., 2004), social support (Westmaas & Langsam, 2005; Pennanen et al., 2014), those with comorbid conditions (Brady, 2020), racial, and ethnic groups (Kulak et al., 2016; Nollen et al., 2021; Trinidad et al., 2011). Therefore, available treatment options may not function effectively for all individuals in a diverse population. These differences offer motivation for additional pharmacogenomic efforts towards creating effective smoking cessation treatments. Individuals in underrepresented socioeconomic groups face additional social challenges in accessing cessation treatment including poor health literacy (Stewart et al., 2013), lack of insurance (Kiefe et al., 1998; Brady, 2020), and other comorbid substance use (Streck et al., 2020) which may also influence research participation. Consequently, there have been calls to increase the diversity of research studies to determine the influence of genetic and social environmental variation on pharmacological response (El-Boraie & Tyndale, 2021) To date, consideration of racial, ethnic, and ancestral diversity in pharmacogenetic research has been most frequently addressed. Reflection and discussion of the current status of pharmacogenetic research from the perspective of race, ethnicity, and ancestry may offer guidance in the future development of effective pharmacogenetic smoking cessation programs in other areas.

Most pharmacogenetic and pharmacogenomic studies of smoking have been conducted in samples of self-identified White race participants (Quaak et al., 2009; Zhang et al., 2019). A common limitation of these results is that they are likely to be not applicable to all racial populations, ethnic groups, nor reflect genetic diversity across ancestry groups (Peterson et al., 2019; Zhang et al., 2019). Failure to account for these differences can lead to the development of pharmacologic treatment strategies for nicotine dependence that do not perform equally well in all smokers making a quit attempt (Goodman & Brett, 2021; Nollen et al., 2021). This in turn may exacerbate health disparities in smoking cessation across racial and ethnic groups (Parker & Satkoske, 2012; Smart et al., 2004). Genomic studies of the pharmacological responses for smoking cessation are beginning to disaggregate the societal contexts of race and ethnicity from the differences in allele distribution across genetic loci by including data across multiple genetic ancestry groups.

While the meaning of *genetic ancestry* can vary by context (Mathieson & Scally, 2020), here we define it to refer to the categorization of participants by patterns of genetic similarity across members of a population. These patterns take advantage of differing allele frequencies which result from several factors, including common geographical origins and large-scale historical events experienced by prior generations (Peterson et al., 2019). Consequently, estimates of genetic ancestry attempt to capture the genetic similarities shared between individuals that may not include the societal complexities of race and ethnicity (Olson et al., 2005; Mersha & Abebe, 2015). Here, we will refer to the most common genetic ancestry groups as currently reported in the Genome Aggregation Database (gnomAD v2.1): African (AFR), Ashkenazi Jewish, East Asian, European (EUR), Latino/Admixed American, Other, and South Asian (Karczewski et al., 2020). Classification of these categories vary as a function of the statistical methods and global reference groups used to categorize study sample participants (Peterson et al., 2019).

Compared to genetic ancestry, race and ethnicity are variables that largely reflect social context. Self-reported *race* has historically been defined through physical appearance and biogeographical ancestry (e.g., country of origin for a study participant’s descendants) which may interact with genetic factors to influence risk for health outcomes (Peterson et al., 2019). Here, self-reported race refers to the categories that society has established to categorize groups of people based on physical characteristics such as hair color and as well as skin pigmentation. Race has become an important factor in health because distribution of risks and opportunities has historically been determined by these categorizations (Jones, 2001). Similarly, self-reported *ethnicity* reflects a “complex multidimensional construct” that is identified by many factors including shared culture, historical influence, social class, shared beliefs, biological background (e.g., genetic factors, descent, or appearance), language, and shared customs (Mersha & Abebe, 2015; Flanagin et al., 2021) Definitions of race and ethnicity can vary between countries (Williams et al., 2010). One example of such categorizations are those used in the United States federal Office of Management and Budget which uses five categories of race (African American/Black, American Indian or Alaska Native, Asian, Native Hawaiian or Other Pacific Islander, and White) and two ethnicity categories (Hispanic/Latino and Not-Hispanic/Not-Latino).

This scoping review is guided by two major research questions “To what degree are individual racial, ethnic, and genetic ancestry groups represented in the existing published literature across pharmacogenetic studies of smoking cessation treatments?” and “Have any pharmacogenetic association results been consistently identified across treatments and racial, ethnic, or genetic ancestry groups?” These questions are addressed because the degree to which current pharmacogenetic study results of smoking cessation applies to all populations remains unclear. Further, it is crucial to establish the current landscape of access to and clinical implementation of pharmacogenetic smoking cessation research across underrepresented populations in order to reduce the likelihood of further exacerbating health inequities. Therefore, we also review the current state of study ascertainment, study design, and statistical analytic strategies to address the inclusion of race, ethnicity, and ancestry data in pharmacogenetic smoking cessation research. The use of a scoping review approach is ideal to identify gaps in knowledge by examining the degree to which existing pharmacogenetic studies of smoking represent various racial, ethnic, and ancestry groups. We will also discuss current trends in conducting pharmacogenomics research and explore opportunities and challenges towards increasing diversity in pharmacogenomic research for smoking cessation. This includes identifying practical barriers in smoking cessation treatment and conducting related research in diverse populations.

## Methods

### Data sources and eligibility criteria

The scoping review was guided by the PRISMA extension for scoping reviews (PRISMA-ScR) (Tricco et al., 2018). A literature search was performed to identify papers published between 1988–2022 in PubMed. We focused on PubMed because it is the primary database that indexes medical literature including pharmacogenetic studies. The search was conducted on 12 October 2022. Inclusion criteria were as follows: 1) published in English, 2) conducted in human subjects, 3) focused on an outcome related to smoking abstinence or smoking cessation, 4) included use of a known pharmacological treatment for nicotine dependence, and 5) used methods testing genetic association. Additionally, the reference lists of four review articles that were found and excluded due to publication type during our PubMed search (Bergen et al., 2015; Ma et al., 2016; Salloum et al., 2018; El-Boraie & Tyndale, 2021) were manually searched to identify any further studies not captured by PubMed search. Although an *a priori* review protocol was developed by the research team, it was not publicly registered in advance.

### Search strategy

The search was focused on articles which satisfied four distinct concepts, each with unique search terms. First, a concept reflecting *pharmacogenomic research* was created which represented the use of pharmacogenomic study design principles. This concept was utilized to identify studies focused on reporting individual response to drug action while accounting for genotype. This concept used the terms “pharmacogenomic” or “pharmacogenetic” or “drug response” or “side effects” or “adverse effects”. A second concept was created to capture *pharmacological smoking cessation methods* currently used by treatment providers. This concept represented the outcome variable or experimental grouping of individuals in the case of clinical trials. This was captured using the following terms: “bupropion”, “varenicline, “nortriptyline”, “cytisine”, “smoking cessation”, “smoking abstinence”, “smoking withdrawal symptoms”, and “former *versus* current smoker”. The third concept was created to examine underrepresented groups in genetically-informative studies. A search term was created to examine groups underrepresented in research (seeded with “Hispanic”, “Asian”, “Latino”, “Native Alaskan”, “Native Hawaiian”, “Pacific Islander”, “Native American”, “Indigenous”, “First Nation”, “education”, “income”, “socioeconomic status”, “LGBTQIA”, “mental health”, “physical disability”, or “cognitive delay”). The fourth concept focused on genetically-informative study designs (“GWAS”, “polygenic score”, “candidate gene”, or “genome-wide association”, “gene”, “genetic”, “genetic association”, and “genotype”). This study focuses on racial, ethnic, and ancestry groups because no published studies were found for other categories of underrepresented populations (e.g., low-income). Each of the concepts were linked in the search with the Boolean operator “AND”. Search terms within concepts were linked using “OR” operators.

### Title and abstract relevance screening

Titles and abstracts from articles were reviewed by five authors (JC, EPW, JW, AE, MS). Each title and abstract were reviewed by at least two authors; any disagreements between reviewers were shared and discussed as a group to arrive at consensus.

### Data extraction

All articles determined to be relevant after title and abstract screening were obtained for subsequent review of the full-text article. Full publications were reviewed and assessed by one team member (AE, BJ, MS, SS, SB, JC, KD, EPW, and JW) and the information extracted was confirmed by one of the first authors (JW or EPW).

A form was developed by the authors to confirm relevance and to extract study characteristics including publication title, first author, year, population description (size, race/ethnicity distribution, and sample ascertainment), publication type, epidemiological study design (i.e., randomized controlled trial, cohort), genetic study design (i.e., GWAS, candidate gene), cessation outcome measured (e.g., smoking abstinence, nicotine metabolite ratio), treatments investigated (including information about psychological/behavioral treatments, number of and duration of treatment arms, drug dose amount and comparison group used), genes investigated, type of genetic association (genotype association only, gene-by-treatment, or both), handling of race and treatment arms in statistical analysis (i.e., pooled and adjusted, stratified), and statistically significant results (see Supplementary Text 1). This form was reviewed by the research team and tested by all reviewers before implementation, resulting in minor modifications. All citations were imported into the web-based review software Rayyan (Ouzzani et al., 2016) for title and abstract screening. Duplicate citations were manually removed.

### Analyzing the data

Quantitative results across studies were synthesized to summarize themes and trends across studies without conducting a meta-analysis (Campbell et al., 2020). Studies were reviewed to address five themes: distribution of studies conducted by racial, ethnic, and/or ancestry groups; genetic study designs used; epidemiological study designs used; treatments studied; adverse events; and associations between genetic variants and cessation outcomes across racial, ethnic, or ancestry groups.

## Results

**FIGURE 1:**
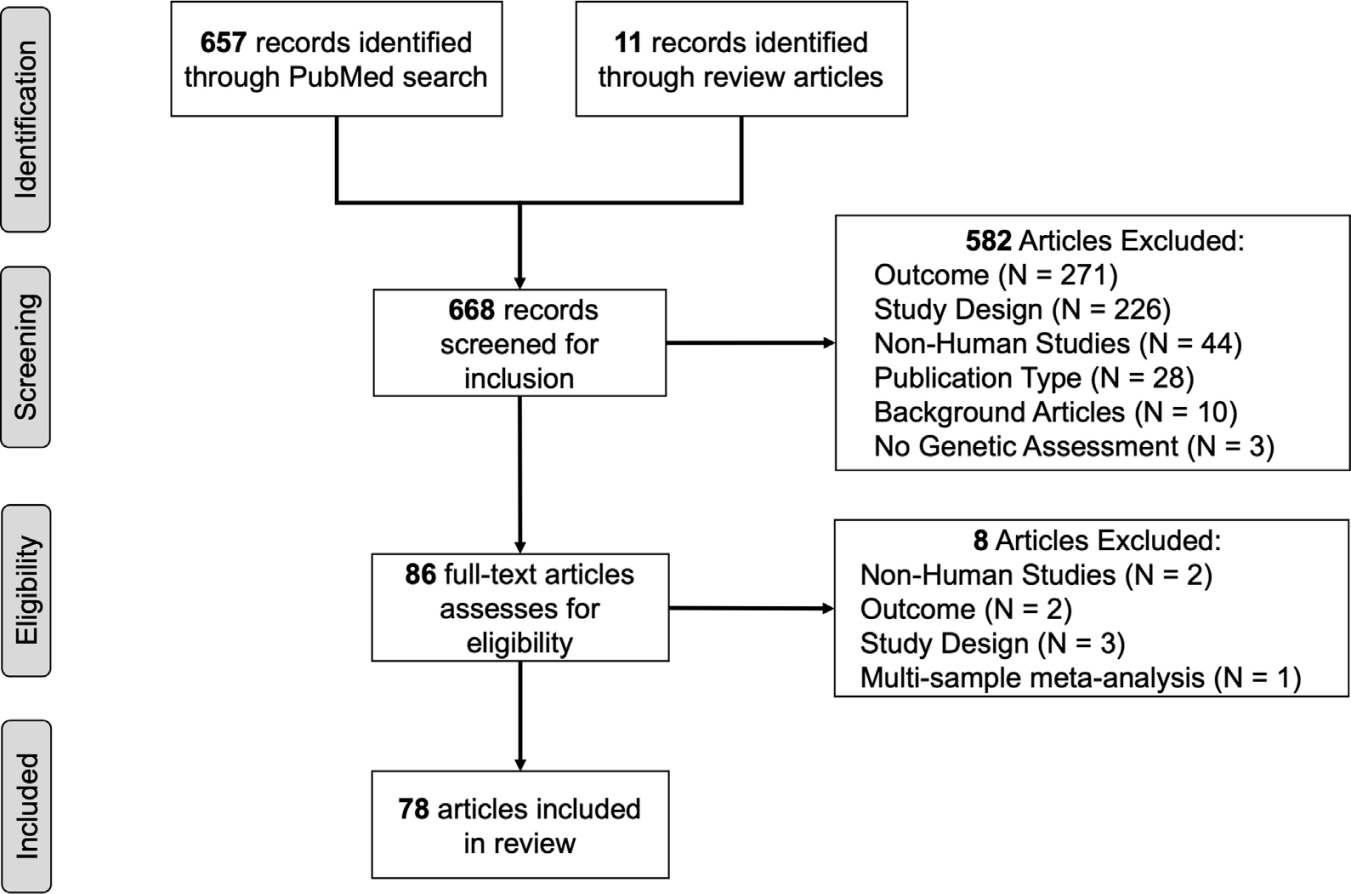
Flowchart of the review process.

The PubMed search produced 657 articles. An additional 11 articles were added after review of the reference lists of review articles (Bergen et al., 2015; Ma et al., 2016; Salloum et al., 2018; El-Boraie & Tyndale, 2021). Articles were excluded because: 1) smoking cessation was not the reported outcome (N = 271) and was often treated as a covariate, 2) used a study design that that did not align with review criteria (i.e., there was no pharmacogenetic component of the study, N = 226), 3) used non-human study designs (e.g., mouse models, N = 44), 4) did not measure genetic variants (N = 3), or 5) for other reasons (3 published in a foreign language, 3 conference paper abstracts, 4 commentary articles, N = 10). This produced 86 articles for full-text review. Seven studies were removed during full article review because incorrect study designs (e.g., a focus on the use of mouse models, meta-analysis, or outcome not focused on smoking abstinence). Results from a final total of 79 articles are summarized (Figure 1)) (Bergen et al., 2014; Breitling et al., 2010; Chen et al., 2012; Cameli et al., 2018; Chen et al., 2014; Chen et al., 2015; Cinciripini et al., 2004; Dahl et al., 2006; David et al., 2007b; David et al., 2007a; David et al., 2008b; David et al., 2008a; David et al., 2013; El-Boraie et al., 2020; Glatard et al., 2017; Gold et al., 2012; Guo & Heitjan, 2010; Heitjan et al., 2008b; Ho et al., 2009; Hutchison et al., 2007; Johnstone et al., 2004; Johnstone et al., 2007; Lee et al., 2007; Lee et al., 2012; Lerman et al., 2002; Lerman et al., 2003; Lerman et al., 2004; Lerman et al., 2006; Lerman et al., 2010; Leventhal et al., 2012; Munafò et al., 2007; Munafò et al., 2009; Munafò et al., 2013; Quintana & Conti, 2013; Robinson et al., 2007; Rocha Santos et al., 2015; Roche et al., 2019; Rose et al., 2010; Swan et al., 2005; Swan et al., 2007; Swan et al., 2012; Tomaz et al., 2015; Tomaz et al., 2018; Tomaz et al., 2019; Santos et al., 2020; Ton et al., 2007; Tyndale et al., 2015; Uhl et al., 2008; Verde et al., 2014; Ware et al., 2015; Zhu et al., 2012; Zhu et al., 2014a; Zhu et al., 2014b) and 18 (23%) estimated genetic ancestry categories (Hu et al., 2006; Berrettini et al., 2007; Ray et al., 2007; Conti et al., 2008; Uhl et al., 2010; Sarginson et al., 2011; King et al., 2012; Bergen et al., 2013a; Ashare et al., 2013; Bergen et al., 2013b; Bergen et al., 2015; Bress et al., 2015; Chen et al., 2018a; Chenoweth et al., 2018; Chenoweth et al., 2021; Chenoweth et al., 2022; Chen et al., 2020; El-Boraie et al., 2021).

Articles were sorted and summarized across multiple themes and summarized below. A summary of overall results from references sorted by genetic epidemiological study design (Supplementary Table S1), epidemiological study design (Supplementary Table S2) and treatment (Supplementary Table S3) are presented below. Additionally, studies were summarized as: genetic study design used by race, ethnicity, and ancestry groups; epidemiological study design used by race, ethnicity, and ancestry groups; and treatment studied by race, ethnicity, and ancestry groups (Supplementary Table S4).

### Overall characteristics of included studies by study country, racial groups, ethnic groups, and genetic ancestry populations

**FIGURE 2:**
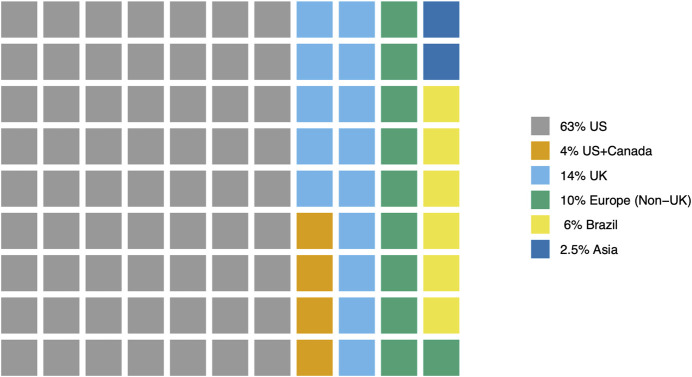
Distribution of reviewed articles by study country.

Of the 79 reviewed articles, the majority of studies (62) were conducted in the United States (US, 63%) and the United Kingdom (United Kingdom, 14%). An additional 8 studies (10%) were conducted in European nations (e.g., Belgium, Germany, Spain) or Canada. Five studies (6%) were conducted in Brazil. Two studies (2.5%) were conducted in Asian nations (China and Korea). Three studies (3.8%) were conducted in two nations- US and Canada (Figure 2).

**FIGURE 3:**
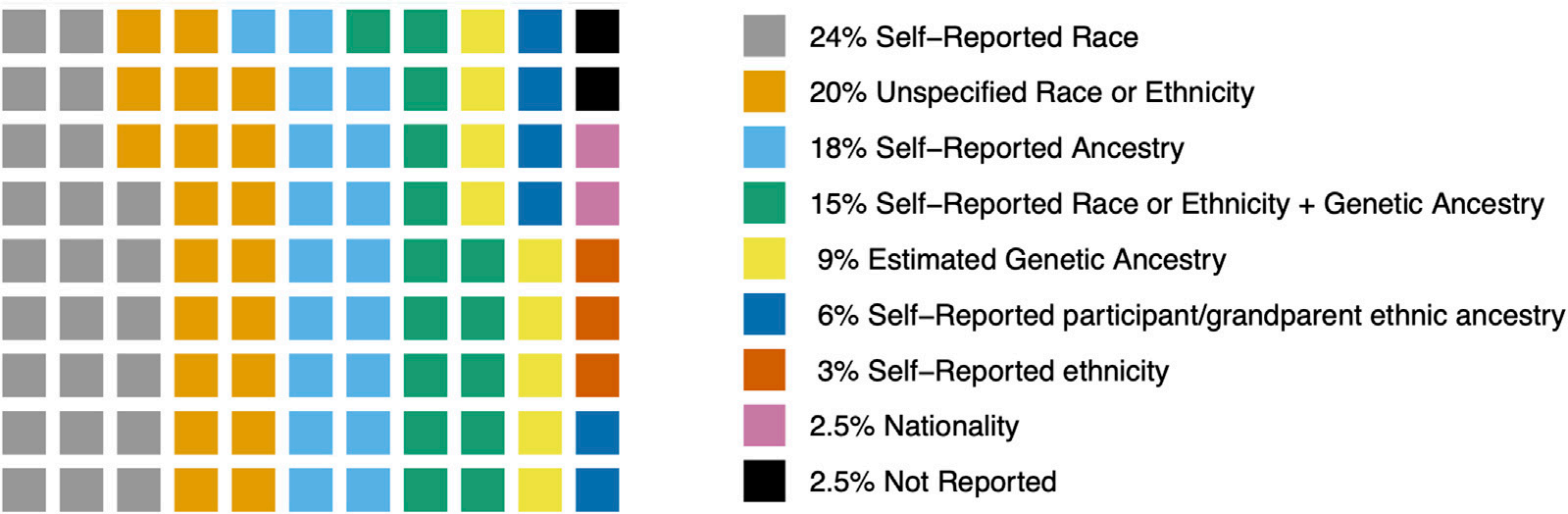
Distribution of the methods used to assign race, ethnicity, and ancestry.

Across all studies, most (19 studies, 24%) used self-reported race. This generally reflects US categories of race (e.g., African American, American Indian and Native Alaskan, Asian Pacific Islander, Black, Caucasian, Hispanic, Other, or White). Sixteen studies (20%) did not specify details on how race, ethnicity, or ancestry was measured. Fourteen studies (18%) measured self-reported ancestry which was generally categorized as European or non-European. Twelve studies (15%) used a measure of self-reported race or ethnicity and further categorized participants into genetic ancestry groups using Ancestry Informative Markers (AIM) or genome-wide association study (GWAS) data (e.g., African- AFR and European- EUR). Seven studies (9%) used AIM or GWAS data to estimate genetic ancestry and classify participants accordingly. Five studies (6%) used self-report of ethnic ancestry of participants or their grandparents (e.g., European, Caucasian, White European, or European Caucasian). Two studies (3%) reported self-report ethnicity (e.g., African American, Asian, Caucasian, Hispanic, Native American, Other, Pacific Islander, or White). Two studies (2.5%) were conducted in European nations (Italy and Germany) and relied on nationality or residence status as an indicator of race or ethnicity (Breitling et al., 2010; Pintarelli et al., 2017) (Table 2). Two studies (2.5%) did not measure or report race, ethnicity or genetic ancestry. One study took place in Korea (Han et al., 2008). One study took place in the Netherlands (Quaak et al., 2012) (Figure 3).

Twenty-five publications (32%) included participants representing non-White race, non-White ethnicity, or non-European ancestry populations (Table 1). These studies reported race, ethnicity, or ancestry using: 1) Self-reported race, 2) Self-reported race or self-reported ethnicity alongside estimated genetic ancestry assessed using ancestry informative marker data (AIM) or genome-wide association study (GWAS) data, 3) Unspecified details of the assessment of race or ethnicity, 4) Estimated genetic ancestry with ancestry informative markers (AIM) or GWAS data, and 5) Self-reported ancestry. One study did not measure race, ethnicity, or ancestry. Three studies used self-reported race to conduct studies exclusively in participants who identified as African American or Black (Ho et al., 2009; Zhu et al., 2012; Zhu et al., 2014b). Two studies used GWAS-based principal components to identify African ancestry (AFR) populations and conduct a single-group association study (Chenoweth et al., 2018; Chenoweth et al., 2022). Two studies reflect a homogeneous group of participants without details on race, ethnic, or ancestry measurement-one study with Han Chinese participants and one study conducted in Korea. Eighteen studies included data from more than one racial, ethnic, or ancestry group. Eight of these multigroup studies included race, ethnicity, or ancestry in their analyses as a covariate to account for the variance due to population stratification on any statistically significant genetic associations. The sample sizes of these multigroup studies (N < 2,000) are too small to make strong conclusions about genetic association results in any one population. Consequently, genetic association results from these studies are summarized as “multigroup studies” and no population-based differences by race, ethnicity, or ancestry are inferred. Genetic association results from studies including participants representing non-White race, non-White ethnicity, or non-European ancestry populations are summarized in Table 3.

**TABLE 1:** Summary of studies including non-white or non-European ancestry participants by treatment

Author	Year	Epidemiologic Study Design	Treatment(s)	Genetic Study Design	Study Country	Total N	Reported Race, Ethnicity, and/or Ancestry* (N)	Significant Genetic Associations
Berretitini et al.	2007	Double-Blind Randomized Placebo- Controlled Trial	BUP	Candidate	USA	511	Self-report race AA (81) EA (430) AIM-based ancestry estimated in EA participants eur	*COMT* (rs737865 and rs165599 haplotype in EA and AA)
Zhu et al.	2012	Double-Blind Randomized Placebo- Controlled Trial	BUP	Candidate	USA	534	Self-report race AA (534)	*CYP2B6* (alleles: *4 K262R, *5 R487C, *6 K262R & Q172H,*9 Q172H, *16, *18, *22)
Han et al.	2008	Open Clinical Trial	BUP	Candidate	Korea	225	Not reported (225)	*DRD2* (Taql A) COMT (Val158Met) NET-8 (A1287G)
Chenoweth et al.	2022	Randomized Placebo- Controlled Trial	BUP	Candidate	USA	173	GWAS-based principal components analysis afr (173)	N/A
O'Gara et al.	2007	Clinical Trial	BUP and NP	Candidate	UK	578	Unspecified European ancestry (502) African ancestry (72)	*DAT1* (3'UTR VNTR and intron 8 VNTR)
Sarginson et al.	2011	Open Label and Double- Blind Randomized Placebo-Controlled Trial	BUP and NP	Candidate	USA	571	Self-report ethnicity AA (19) Asian (33) Caucasian (442) Hispanic (52) Native American (4) Other (17) Pacific Islander (4) AIM-based principal components analysis*	*CHRNA5* (rs16969968 and rs1051730)
Tomaz et al.	2015	Cohort Study	BUP, NP, VAR	Candidate	Brazil	478	Self-report race Non-White (331) White (147)	*CYP2B6* (rs2279343)
Tomaz et al.	2018	Cohort Study	BUP, NP, VAR	Candidate	Brazil	1049	Self-report race Asian (31) Amerindians (1)	*CHRNA5* (rs2036527 and rs16969968)
Chen et al.	2020	Genotype-Stratified Double Blind Randomized Placebo- Controlled Trial	cNRT and VAR	Candidate	USA	826	Self-report race Black/AA Ancestry (270) Other (36) White/European Ancestry (516)	N/A
Ton et al.	2007	Double-Blind Randomized Placebo- Controlled Trial	d,1-fenfluramine	Candidate	USA	593	Self-report race AA (20) American Indian & Native Alaskan (3) Asian Pacific Islander (5) Caucasian American (550) Hispanic/Latin American (2) Other (13)	N/A
Ho et al.	2009	Double-Blind Randomized Placebo- Controlled Trial	Gum	Candidate	USA	495	Self-report race AA/Black (495)	*CYP2A6* (alleles: *1B, *4, *9,*17, *20, *27, *28, *35)
Sun et al.	2012	Double-Blind Randomized Placebo- Controlled Trial	Lozenge	Candidate	China	230	Unspecified Han Chinese (230)	*COMT* (Val108Met)
Roche et al.	2019	Double-Blind Randomized Placebo- Controlled Trial	Naltrexone with NP	Candidate	USA	280	Self-report race AA (99) Caucasian (163) Other (18)	N/A
Zhu et al.	2014b	Double-Blind Randomized Placebo- Controlled Trial	Study 1: BUP	Candidate	USA	Study 1: 42	Self-report race Study 1: Asian (7), AA (14), Caucasian (21) Study 2: AA (540)	Study 1: *CYP2C19* (*1/*1;*1/*2; *1/*17, *2/*17)
			Study 2: BUP			Study 2: 540		
Zhu et al.	2014a	Double-Blind Randomized Placebo-Controlled Trial	Study 1: Gum	Candidate	USA	Study 1: 609	Self-report race Study 1: AA (609) Study 2: AA (534)	Study 1: *CHRNA5-A3-B4* (rs2036527, rs16969968, and rs588765) Combined: *CHRNA5-A3-B4* (rs2036527 and rs588765)
			Study 2: BUP			Study 2: 534		
Tomaz et al.	2019	Cohort Study	VAR	Candidate	Brazil	167	Self-report race Non-White (27) White (140)	*CYP2B6* (rs8109525)
Santos et al.	2015	Cohort Study	VAR, NP, BUP	Candidate	Brazil	483	Self-report race Non-White (181) White (302)	*CHRNA4* (rs1044396)
Santos et al.	2020	Cohort Study	VAR	Candidate Gene Expression	Brazil	27	Self-report race Non-White (6) White (21)	*CHRNA7* gene expression and *CHRNG* gene expression
El-Boraie et al.	2021	Randomized Placebo-Controlled Trial	VAR, NP Replicated in NP, VAR, and BUP	Candidate Genetic Risk Score	USA	GRS: 1887 Replication: 412	GWAS-based ancestry GRS: AFR (954), EUR (933), admixed AFR & EUR (68) Replication: AFR (216), EUR (196)	*CYP2A6* (rs12459249, rs111645190, rs185430475, *1x2 Duplication, *4 Deletion,*9 rs28399433, *12 Hybrid, *17 rs28399454, *20 rs568811809,*25/*26/*27 rs28399440, *35 rs143731390)
Bress et al.	2015	Double-Blind Randomized Placebo-Controlled Trial	Naltrexone with NP	Genetic Ancestry Score	USA	231	Self-report race Non-Hispanic AA (95) Caucasian American (136) AIM-based ancestry West African Ancestry	Low West African Ancestry
Chenoweth et al.	2018	Randomized Placebo-Controlled Trial	NP, gum, VAR, BUP	GWAS	USA & Canada	954	Self-report race AA (954) GWAS-based ancestry AFR	96 significant associations. Most important- SNPs near *CYP2A6* (rs12459249, rs111645190)
Rose et al.	2010	Double-Blind Randomized Placebo-Controlled Trial	NP	Quit Success Genetic Risk Score	USA	467	Self-report race AA (87) European American (358) Other (22) GWAS-based ancestry AFR, EUR	N/A
Munafo et al.	2013	Randomized Clinical Trial	NP + NRT	Candidate	UK	598	Self-report ancestry European ancestry (547) Non-European ancestry (51)	N/A
Robinson et al.	2006	Double-Blind Randomized Placebo-Controlled Trial	Venlafaxine	Candidate	USA	265	Unspecified ethnicity European (243) Other (22)	N/A
Cinciripini et al.	2004	Double-Blind Randomized Placebo-Controlled Trial	Venlafaxine + NRT	Candidate	USA	134	Unspecified ethnicity European (123) Black (6) Hispanic (4) Other (1)	*DRD2* (Taql A)

AIM = Ancestry Informative Markers, GWAS = Genome-wide Association Study, BUP = Bupropion, VAR = Varenicline, NRT = Nicotine Replacement Therapy, cNRT = conbinded NRT, NP = Nicotine Patch, AA = African American, EA = European American, Nations = USA and UK, ASW = AA from Southwest of US, CEU = UTAH resident of northwestern European ancestry, MEX = Mexian Americans living in Los Angeles, CHD = Chinese living in Metropolitan Denver, TSI = Tuscans, Italy

Of the 79 reviewed articles, 54 studies (68%) exclusively included participants representing White race, European/Caucasian/White ethnicity, or European/EUR ancestry populations (Table 2). In addition to the aforementioned definitions used in studies including participants representing non-White race, non-White ethnicity, or non-European ancestry population, these studies assessed race, ethnicity, or ancestry as: 1) Self-reported ethnic ancestry of participants or their grandparents and 2) Self-reported ethnicity. Two multigroup studies included data with 5% or fewer participants from racial groups classified as Non-White or Other (Johnstone et al., 2004; Glatard et al., 2017). These studies have been defined as White race studies because the variation due to the other racial groups is not expected to influence genetic association results. Genetic association results from these studies are summarized according to how race, ethnicity, or ancestry were reported by study authors in Table 4.

**TABLE 2:** Summary of studies including white or European ancestry participants by treatment

Author	Year	Epidemiologic Study			Treatments		Genetic	Study		Reported Race		Significant Genetic Associations
David et al.	2007a	Double-Blind Randomized Placebo-Controlled Trial			BUP		Candidate	USA		Self-report ethnic ancestry European (291)		*DRD2* (Taql A) and *CYP2B6* (exon 9 1459 C>T)
Conti et al.	2008	Double-Blind Randomized Placebo-Controlled Trial			BUP		Candidate	USA		Self-report ethnicity Caucasian AIM-based ancestry eur (412)		End of Treatment: *ADCYAP1* (rs12961210), *CHRNA2* (rs2565059), *CHRNB2* (rs2072661 and rs2072660), *HTR1B* (rs1936158), *TDO2* (rs10517626)
												6 Month Follow-up: *CDK5* (rs2069454), *CHRNB2* (rs2072661, rs2072660, rs1127314, rs2131902, and rs3766927), *FOSB* (rs2238687), *TDO2* (rs10517626 and rs13152449)
Leventhal et al.	2012	Double-Blind Randomized Placebo-Controlled Trial			BUP		Candidate	USA		Self-report ethnic ancestry White European (331)		N/A
David et al.	2013	Double-Blind Randomized Placebo-Controlled Trial			BUP		Candidate	USA		Self-report racial/ ethnic heritage		*DRD4* (VNTR)
										White, non-Hispanic (792)		
Bergen et al.	2013	Double-Blind Randomized Placebo-Controlled Trial			BUP		Candidate	USA		Self-report race White AIM-based ancestry eur (416)		*DRD4* (VNTR)
Swan et al.	2007	Open Label Randomized Trial			BUP		Candidate	USA		Self-report ethnicity White (323)		*DRD2* (TaqlA) and *SLC6A3* (VNTR)
Swan et al.	2005	Randomized Effectiveness Trial			BUP		Candidate	USA		Self-report ethnicity White (416)		*DRD2* (Taql A)
Lerman et al.	2002	Randomized Placebo-Controlled Trial			BUP		Candidate	USA		Self-report ethnic ancestry of grandparents European Caucasian (426)		*CYP2B6* (exon 9 1459 C>T)
Lerman et al.	2003	Randomized Placebo-Controlled Trial			BUP		Candidate	USA		Self-report ethnic ancestry of grandparents European Caucasian (418)		*DRD2* (Taql A) and *SLC6A3* (VNTR)
Hu et al.	2006	Double-Blind Randomized Placebo-Controlled Trial			BUP		Candidate	USA		Unspecified		N/A
										White (553)		
Lee et al.	2007	Randomized Placebo-Controlled Trial			BUP		Candidate	USA		Self-report ethnic ancestry of grandparents Caucasian (342)		*CYP2B6* (allele: *1/*6)
Heitjan et al.	2008	Randomized Placebo-Controlled Trial			BUP		Candidate	USA		Self-report ancestry European ancestry (436)		N/A
Guo et al.	2010	Randomized Placebo-Controlled Trial			BUP		Candidate	USA		Self-report race (782)		*CHRNA5* (rs871058)
Ashare et al.	2013	Randomized Placebo-Controlled Trial			BUP and cNRT		Candidate	USA		Self-report ancestry & AIM-based ancestry EUR (917)		*APOE* (ε4)
Chen et al. UW-TTRUC	2014	Randomized Placebo-Controlled Trial			BUP and cNRT		Candidate	USA		Unspecified European American (709)		*CYP2A6* (rs16969968 and rs680244 haplotype)
Breitling et al.	2010	Cohort Study			BUP and NP		Candidate	Germany		Self-report nationality (577)		*DRD2/ANKK1* (rs1800497)
King et al.	2012	Double-Blind Randomized Placebo-Controlled Trial			BUP and VAR		Candidate	USA		AIM-based ancestry EUR (1175)		ABSTINENCE AT WEEKS 9-12: Varenicline: *CHRNA4* (rs3787138, rs2236196, and rs6062899), *CHRNA5* (rs518425) *CHRNA7* (rs6494212) *CHRNB2* (rs4292956 and rs3811450), *IREB2* (rs2938674), and LOC123688 (rs7164594) Bupropion: *CHRND* (rs3762528 and rs6725786), *CYP2B6* (rs8109525, rs1808682, rs1042389, and rs2113103) Overall: *CHRNA4* (rs6010918), *CYP2B6* (rs8109525, rs1808682, rs2014141, and rs2113103) ABSTINENCE AT WEEKS 9-52 Bupropion: *CYP2B6* (rs1808682, rs1042389, rs2113103, and rs8100458) Overall: *CHRNA10* (rs7123164), *CYP2A6* (rs7255616), *CYP2B6* (rs1808682 and rs892216), and *HTR3B* (rs11606194)
Gold et al.	2012	Open Label and Randomized Placebo-Controlled Trial			BUP, NP, NS		Candidate	USA		AIM-based ancestry EUR (1217)		*GALR1* (rs2717162)
Bergen et al.	2013	Double-Blind Randomized Placebo-Controlled Trial, 8-Study Mega Analysis			BUP, NP, VAR		Candidate	USA & Canada		Self-report race White AIM-based ancestry eur (2633)		*CHRNA5* (rs588765) and *CHRNA3* (rs1051730)
Quintana et al. PNAT	2013	Randomized Placebo-Controlled Trial			BUP, NP, NS		Candidate	USA		Self-report race White AIM-based ancestry EUR (789)		Significant SNPs using Bayesian Models with Group Bridge, Elastic Net, and Lasso tests of association: *CYP2A6*, *CHRNA7* (rs6494211 rs4779969, and rs16956223), *CHRNA5* (rs3743077), *CHRNB2* (rs2072660 and rs3811450)
Quaak et al.	2011	Randomized Placebo-Controlled Trial			BUP, nortriptyline		Candidate	Netherlands		Not assessed (214)		*5-HTTLPR* (STin2 and rs25531)
Chen et al. UW-TTRUC & Pfizer	2015	Randomized Placebo-Controlled Trial			cNRT and VAR		Candidate	USA		Unspecified European ancestry (1118)		*CHRNA5* (rs16969968)
Johnstone et al.*	2004	Double-Blind Randomized Placebo-Controlled Trial			NP		Candidate	UK		Self-report race Non-White (9) White (746)		*DRD2* (Taql A) and *DBH* (1368)
Johnstone et al.	2007	Double-Blind Randomized Placebo-Controlled Trial			NP		Candidate	UK		Unspecified European ancestry (724)		*COMT* (Val 108/158Met)
Munafo et al.	2007	Double-Blind Randomized Placebo-Controlled Trial			NP		Candidate	UK		Self-report ancestry European ancestry (710)		*OPRM1* (A118G)
David et al.	2007b	Double-Blind Randomized Placebo-Controlled Trial			NP		Candidate	UK		Unspecified European ancestry (722)		N/A
David et al.	2008b	Double-Blind Randomized Placebo-Controlled Trial			NP		Candidate	UK		Unspecified European ancestry (720)		*DRD4* (VNTR)
Lerman et al.	2010	Double-Blind Randomized Placebo-Controlled Trial			NP		Candidate	USA		Unspecified Caucasian (471)		*CYP2A6* (alleles: *2, *9, *12, and *4)
Munafo et al.	2009	Randomized Clinical Trial			NP		Candidate	UK		Self-report ancestry European (804)		N/A
David et al.	2008a	Randomized Placebo-Controlled Trial			NP		Candidate	UK		Self-report ancestry European ancestry (792)		N/A
Ruyck et al.	2010	Randomized Placebo-Controlled Trial			NP		Candidate	Belgium		Unspecified European ancestry (233)		N/A
Lerman et al.	2004	Open Label Randomized Placebo-Controlled Trial			NP and NS		Candidate	USA		Self-report ancestry European ancestry (320)		*OPRM1* (Asn40Asp)
Verde et al.	2014	Open Label Randomized Trial			NP, BUP		Candidate	Spain		Unspecified European race (70)		NP group: *5-HTTLPR* and *CYP2A6* (*2, *9, *12,*1x2)
												BUP group: *CYP2A6* (*2, *9, *12, *1x2)
Ware et al.	2015	Randomized Placebo-Controlled Trial			NP, Gum		Candidate	UK		Self-report ancestry European ancestry (448)		N/A
Ray et al.	2007	Open Label Randomized Trial			NP, NS		Candidate	USA		Self-report ancestry European ancestry (374)		*OPRMI1* (Asn40Asp)
												*HINT1* (rs3852209)
Hutchison et al.	2007	Open Label Randomized Trial			NP, NS		Candidate	USA		Self-report ancestry/race European ancestry (316)		*CHRNA4* (rs2236196)
Tyndale et al.	2015	Randomized Placebo-Controlled Trial			NP, VAR		Candidate	USA & Canada		Unspecified Caucasian (654)		N/A
Bergen et al.	2014	Randomized Placebo-Controlled Trial			NP, VAR, BUP		Candidate	USA		Self-report ancestry European ancestry (1839)		End of Treatment: *SLC22A2* (rs316006) 6 Month Follow-up: *SLC22A2* (rs316006 and rs316019)
Dahl et al.	2006	Open Label Randomized Trial			NS, NP		Candidate	USA		Self-report race European (363)		*DRD2* (-141C Ins/Del)
Lerman et al.	2006	Double-Blind Randomized Placebo-Controlled Trial			Study 1: BUP		Candidate	USA		Study 1: Self-report race European (414)		Study 1: End of Treatment: *DRD2* (-141C Ins/Del) 6 Months After Treatment: *DRD2* (C957T)
					Study 2: NP, NS					Study 2: Self-report race European (368)		Study 2: End of Treatment: *DRD2* (-141C Ins/Del) and *DRD2* (C957T)
Lee et al.	2012	Study 1: RCT			Study 1: BUP		Candidate	USA		Study 1: Self-report race & AIM-based ancestry EUR (411)		Males-Only (across treatments): End of Treatment: *CHRNA3-CHRNA5* (rs578776) and *EPB41* (rs6702335, rs12021667, rs1207267, rs12039988, rs203278, rs150089, rs2985322, rs4654390, rs10915216) 6 Month Follow-up: *CALY* (rs11101694), *EPB41* (rs6702335, rs12021667, rs1207267, rs12039988, rs168237840), and *FOSB* (rs2238687)
		Study 2: Open Label Randomized Trial			Study 2: NP, NS					Study 2: Self-report race & AIM-based ancestry EUR (378)		Females-Only (across treatments): End of Treatment: *ANKK1* (rs7123797, rs4938012, rs4938012, and rs17115439), and *CHRNA4* (rs4809549) 6 Month Follow-up: *CHRNB2* (rs3766927, rs1127314, rs2131902), *CLIC6* (rs2834600), and *MAPK1* (rs17759598)
Chen et al.	2012	Study 1: Cross-sectional			Study 1: No Treatment		Candidate	USA		Study 1: Self-report ancestry European ancestry (5,216)		Study 2: *CHRNA5-CHRNA3-CHRNB4* (rs16969968 and rs680244 haplotypes)
		Study 2: Randomized Placebo-Controlled Trial			Study 2: BUP, Nicotine Lozenge, NP, cNRT					Study 2: Self-report ancestry European ancestry (1,073)		
Cameli et al.	2018	Case-Control			VAR		Candidate	Italy		Unspecified European (142)		*CHRFAM7A* (exon 6 2bp repeat copy number)
Swan et al.	2012	Randomized Clinical Effectiveness Trial			VAR		Candidate	USA		Self-report race European (397)		N/A
Pintarelli et al.	2017	Retrospective Cohort Study			VAR, BUP, NP		Candidate	Italy		Unspecified Italian residence		*CHRNA5* (rs503464)
										European (337)		
Chen et al.	2018	Randomized Placebo-Controlled Trial			BUP, Lozenge, NP		Candidate Genetic Risk Score	USA		GWAS-based principal component analysis EUR (1067)		N/A
El-Boraie et al.	2020	Randomized Placebo-Controlled Trial			Study 1: NP, VAR		Candidate Genetic Risk Score	USA		Study 1: AIM-based ancestry EUR (933)		*CYP2A6* (rs56113850, rs2316204, rs113288603, *2 rs1801272, *9 rs28399433, *4 deletion, *12 hybrid)
					Study 2: Gum, NP, BUP					Study 2: AIM-based ancestry EUR (193)		
Uhl et al.	2010	Randomized Placebo-Controlled Trial			NP		GWAS	USA		Unspecified European (369)		*DIP2C* (rs12245224), *MCF2L2* (rs9882117), *OR2AP1* (rs2371189), *PSD3* (rs6992325), *UPS13* (rs4854948), *WDR72* (rs1995318), Intergenic (rs17090633, rs1348637, rs10023214, rs4677135, rs372412, rs10252483, rs763980, rs10475190)
Uhl et al.	2010	Randomized Placebo-Controlled Trial			NP		GWAS	UK		Unspecified European (925)		N/A
Chenoweth et al.	2021	Randomized Placebo-Controlled Trial			VAR, NP		GWAS	Canada		AIM-based ancestry European ancestry (1246)		Genome-wide Significant Variants: *SLCO3A1* (rs1568209)
Glatard et al.	2017	Cohort Study			NP and VAR		Candidate	Switzerland		Self-report race European(147) Other (6)		*CYP2A6* (rs1801272 and rs28399433)
Uhl et al.	2008	Double-Blind Randomized Placebo-Controlled Trial			BUP, NP, NS		Poooled GWAS	USA		Self-report race European(550)		*CLSTN2*, *TEK*, *CDH13*, *PTPRT*, *PRKG1*, *ATP9A*, *THSD4*, *PARD3*
Bergen et al.	2015	Study 1: Meta-analysis Randomized Placebo- Controlled Trial			VAR, Various Pharmacotherapy		Study 1: Candidate	USA		Study 1: Self-report ancestry EUR (449)		Study 1: *CYP2A6* (rs4803381, rs1137115, rs4079369, rs8192729), *CYP2D6* (rs1080985, rs28371725, rs16947, rs1080983, rs1065852, rs28360521, rs1800716, rs3892097, rs1135840, rs1058164)
		Study 2: Mega-regression Randomized Placebo- Controlled Trial					Study 2: Candidate			Study 2: Self-report ancestry EUR (2497)		Study 2: *CYP2A6* (rs4803381 and rs1137115)
Turner et al.	2014	Study 1: Open Label Randomized Trial			NP		Study 1: Candidate	USA		Study 1: Self-report ancestry EUR (449)		N/A
		Study 2: Open Label Randomized Trial					Study 2: Candidate			Study 2: Self-report ancestry EUR (174)		

AIM = Ancestry Informative Markers, GWAS = Genome-wide Association Study, BUP = Bupropion, VAR = Varenicline, NRT = Nicotine Replacement Therapy, cNRT = conbinded NRT, NP = Nicotine Patch, AA = African American, EA = European American, Nations = USA and UK, ASW = AA from Southwest of US, CEU = Utah resident of northwestern European ancestry, MEX = Mexican Americans living in Los Angeles, CHD = Chinese living in Metropolitan Denver, TSI = Tuscans, Italy, UW-TTURC = University of Wisconsin Tobacco Use Research Center. *Studies that included Non-European ancestry participants but at less that <5% of total sample size were considered European Only for the purposes of grouping studies.

### Racial, ethnic, and genetic ancestry characteristics across genetic epidemiology study designs

**Candidate gene studies**. Most articles (N = 68, 86%) conducted candidate gene association studies (Table 1; Table 2). These studies most often tested the associations between smoking abstinence and variants located in genes whose products are anticipated to have biological relevance in the following areas: 1) nicotine pharmacokinetics due to the metabolism of nicotine (e.g., *CYP2A6*); 2) pharmacokinetics or pharmacodynamics of the treatments used for smoking cessation (e.g., *CYP2B6* or *CYP2C19* and metabolism of bupropion); 3) nicotine pharmacodynamics related to the neurobiological impacts of nicotine binding to nicotinic acetylcholine receptors and downstream release of the neurotransmitters dopamine, serotonin, and norepinephrine (e.g., *CHRNA3*, *CHRNA*, *CHRNA5*, *CHRNA7*); and 4) neurotransmitter impacts in the presence of smoking cessation treatment (e.g., *DRD2, COMT*, Table 3 and Table 4).

**TABLE 3:** Distribution of studies including Non-White or Non-European participants testing and reporting significant candidate gene association with treatment-related abstinence by reported Ancestry, Race, or Ethnicity assessment method

Gene	Estimated Ancestry^1^ AFR		Self-Report Race^2^ African American		Estimated Ancestry^1^ Multiple Groups		Self-Report Race^2^ Multiple Groups		Unspecified Method^3^ Multiple Groups	
	Tested	Significant	Tested	Significant	Tested	Significant	Tested	Significant	Tested	Significant
*5-HTTLPR*	-	-	-	-	-	-	-	-	-	-
*ANKK1*	-	-	-	-	-	-	-	-	-	-
*APOEe4*	-	-	-	-	-	-	-	-	-	-
*CHRFAM7A*	-	-	-	-	-	-	-	-	-	-
*CHRNA10*	-	-	-	-	-	-	-	-	-	-
*CHRNA2*	-	-	-	-	-	-	-	-	-	-
*CHRNA3*	-	-	1	1	-	-	-	-	-	-
CHRNA4*^	-	-	-	-	-	-	1	1	-	-
CHRNA5*♦^	-	-	1	1	1	1	1	1	-	-
*CHRNA6*	-	-	-	-	-	-	-	-	-	-
*CHRNA7*	-	-	-	-	-	-	-	-	-	-
*CHRNB1*	-	-	-	-	-	-	-	-	-	-
*CHRNB2*	-	-	-	-	-	-	-	-	-	-
*CHRNB3*	-	-	-	-	-	-	-	-	-	-
*CHRNB4*	-	-	1	1	-	-	-	-	-	-
*CHRND*	-	-	-	-	-	-	-	-	-	-
COMT*^	-	-	-	-	1	1	-	-	1	1
*CYP2A6+⧫^*	1	1	1	1	1	1	-	-	-	-
CYP2B6*^	-	-	1	1	-	-	2	2	-	-
*DAT1*	-	-	-	-	-	-	-	-	1	1
*DBH*	-	-	-	-	-	-	-	-	-	-
*DRD2*	-	-	-	-	-	-	-	-	-	-
*DRD4*	-	-	-	-	-	-	-	-	-	-
*GALR1*	-	-	-	-	-	-	-	-	-	-
*HINT1*	-	-	-	-	-	-	-	-	-	-
*HTR3B*	-	-	-	-	-	-	-	-	-	-
*IREB2*	-	-	-	-	-	-	-	-	-	-
*OPRD*	-	-	-	-	-	-	-	1	-	-
*OPRK*	-	-	-	-	-	-	-	1	-	-
*OPRM1*	-	-	-	-	-	-	-	-	-	-
*SLC22A2^*	-	-	-	-	-	-	-	-	-	-
*SLC6A3^*	-	-	-	-	-	-	-	-	-	-
*TH*	-	-	-	-	-	-	1	-	-	-

Reported Ancestry/Race/Ethnicity:

^1^ Genetic Ancestry estimated with AIM or GWAS data

^2^ Race via self-report (e.g., African American, Amerindian, American Indian, Native Alskan, Asian, Asian/Pacific Islander, Black, Caucasian, Hispanic/Latin American, Intermediate, White)

^3^ Race or ethnicity assessment not detailed (Black, Hispanic, European, Other) Bolded gene names indicate a gene identified across multiple studies

*Gene also reported in at least one significant gene-treatment interaction study including non-White participants

+Gene also reported in non-eur GWAS

♦Gene with product influencing nicotine metabolism and pharmacokinetics

^Gene with product influencing medication dosage, effectiveness, and pharmacodynamic interactions

**TABLE 4:** Distribution of studies including only White or European participants testing and reporting significant candidate gene association with treatment-related abstinence by reported Ancestry, Race, or Ethnicity assessment method

Gene	Estimated Ancestry ^1^ EUR		Self-Report Race^2^		Self-Report Ethnic Ancestry^4^		Self-Report Ancestry^5^		Self-Report Ethnicity^6^		Unspecified Method^3^ White/European	
	Tested	Significant	Tested	Significant	Tested	Significant	Tested	Significant	Tested	Significant	Tested	Significant
*5-HTTLPR*	1	1	-	-	-	-	-	-	-	-	2	1
*ANKK1*	1	-	-	-	-	-	-	-	-	-	-	-
*APOEe4*	1	-	-	-	-	-	-	-	-	-	-	-
*CHRFAM7A*	-	-	-	-	-	-	-	-	-	-	1	1
*CHRNA10*	-	-	-	-	-	-	1		-	-	-	-
*CHRNA2*	1	-	-	-	-	-	1		-	-	1	-
*CHRNA3*	2	1	-	-	-	-	3	1	-	-	1	-
CHRNA4*^	1	1	-	-	-	-	2	1	-	-	-	-
CHRNA5*♦^	4	4	1	1	-	-	2	2	-	-	2	1
*CHRNA6*	-	-	1	-	-	-	1		-	-	-	-
*CHRNA7*	2	1	1	1	-	-	1		-	-	-	-
*CHRNB1*	-	-	1	-	-	-	-	-	-	-	-	-
*CHRNB2*	4	1	-	-	-	-	1		-	-	-	-
*CHRNB3*	-	-	1	-	-	-	1		-	-	-	-
*CHRNB4*	1	-	-	-	-	-	1	1	-	-	1	-
*CHRND*	1	-	-	-	-	-			-	-	-	-
COMT*^	-	-	-	-	1	1	-	-	-	-	3	1
*CYP2A6+⧫^*	3	3	1	1	-	-	1	1	-	-	3	3
CYP2B6*^	1	1	-	-	3	3	-	-	-	-	-	-
*DAT1*	-	-	-	-	-	-	-	-	-	-	-	-
*DBH*	-	-	1	1	-	-	-	-	-	-	-	-
*DRD2*	-	-	3	3	2	2	-	-	2	2	2	
*DRD4*	-	-	2	2	1	-	2	1	-	-	1	1
*GALR1*	1	1	-	-	-	-	-	-	-	-	-	-
*HINT1*	1	-	-	-	-	-	1	1	-	-	-	-
*HTR3B*	1	-	-	-	-	-	-	-	-	-	-	-
*IREB2*	1	1	-	-	-	-	-	-	-	-	-	-
*OPRM1*	-	-	-	-	-	-	2	2	-	-	-	-
*SLC22A2^*	-	-	-	-	-	-	1	1	-	-	-	-
*SLC6A3^*	-	-	-	-	1	1	-	-	1	1	-	-
*TH*	-	-	-	-	-	-	-	-	-	-	-	-

Ancestry/Race/Ethnicity:

^1^ Genetic Ancestry estimated with AIM or GWAS data

^2^ Race via participant self-report (White)

^3^ Race or ethnicity assessment not detailed (European or Caucasian)

^4^ Ethnic ancestry of participant or participant’s grandparents via self-report (European or European Caucasian)

^5^ Ancestry via self-report (European)

^6^ Ethnicity via self- report (White). Bolded gene names indicate a gene identified across multiple studies

*Gene also reported in at least one significant gene-treatment interaction study including non-White participants

+Gene also reported in non-eur GWAS

⧫Gene with product influencing nicotine metabolism and pharmacokinetics

^Gene with product influencing medication dosage, effectiveness, and pharmacodynamic interactions.

Gene names from two studies of White participants (Conti et al. 2008 and Lee et al. 2012) that tested associations in loci across 50 genes are not included (see Supplement X)

**Genome-wide association studies**. Five (6%) studies used GWAS to identify loci across the genome that are associated with smoking cessation outcomes. One GWAS was conducted in a sample of African American race/AFR ancestry participants and identified significant associations with several loci near *CYP2A6* (Chenoweth et al., 2018). GWAS in EUR samples reported significant associations with loci across multiple genes (Uhl et al., 2010; Chenoweth et al., 2021) though these have not yet been replicated in other pharmacogenomic studies of smoking cessation.

**Genetic risk scores.** Five studies used genetic risk scores aggregating data from genetic variants with expected associations with quit success (Rose et al., 2010), nicotine metabolism efficiency (via nicotine metabolism ratio) (Chen et al., 2018a; El-Boraie et al., 2020; El-Boraie et al., 2021) and genetic ancestry scores (Bress et al., 2015). Three were conducted in conducted in multiple race, ethnicity, or ancestry group samples (Table 1) and two were conducted exclusively in White race, White ethnicity, or European ancestry samples (Table 2).

**Gene expression and epigenetic studies**. One study that assessed gene expression was identified. This study was conducted in participants representing multiple racial groups in Brazil and who received varenicline (Santos et al., 2020). Gene expression was studied across 17 genes with neurobiological impacts of nicotine binding to nicotinic acetylcholine receptors leading to downstream release of the neurotransmitters (e.g., *DRD1-DRD4, HTR3A, HTR3B, COMT*, and *SLC6A3*). Significant differences in expression were detected for *CHRNA7* and *CHRNG*. No epigenetic studies of pharmacological treatment for smoking cessation in humans were identified for this review.

### Racial, ethnic, and ancestry characteristics across epidemiological study designs

The clinical utility of smoking cessation treatments was assessed across five study designs. Double-blind randomized placebo-controlled trials was the most commonly used study design (29 studies, 37%). This is considered a gold standard approach for evaluating treatment efficacy because it limits various sources of bias, including 1) researcher and adherence bias by blinding assigned treatment groups and 2) treatment selection bias through randomized of participants into treatment groups. Additionally, each treatment group is compared against a placebo group, and improves the estimation differences due to treatment. Three double-blind randomized placebo-controlled trials were conducted in exclusively in African American race or AFR ancestry samples. Ten studies were conducted in multiple race, ethnicity, or ancestry group samples (Table 1). One double-blind randomized placebo-controlled trial was conducted in China among Han Chinese participants. Almost half of these studies (14 out of 29 studies, 48%) included non-White race, non-White/Caucasian ethnicity, non-European ancestry participants. Almost one-third of studies (15 out of 54 studies, 28%) conducted exclusively in White race, White ethnicity, or European ancestry samples used this study design (Table 2).

Twenty-six studies conducted randomized placebo-controlled trials either alone or in combination with open label designs. Randomized placebo-controlled trials share many of same control measures as double-blind randomized placebo-controlled trials. However, only the participants are blind to treatment group assignment. Of these studies, two used data from multiple group samples. Three studies were conducted using this study design in AFR ancestry participants. Twenty-two studies were conducted in samples exclusively of participants representing White race, European/Caucasian/White ethnicity, or European/EUR ancestry populations.

Twelve studies engaged in open label randomized trials, where participants were randomized to treatment groups and were aware of their treatment group assignment. While this accounts for possible treatment selection bias, it does not always control for placebo effects or for the effect of a subject knowing which treatment group they were assigned. Eleven studies included data exclusively of participants representing White race, European/Caucasian/White ethnicity, or European/EUR ancestry populations (Table 2).

Two studies were categorized as “other types of clinical trials”. One study conducted in Korea used the same treatment in all participants (Han et al., 2008). Another study conducted in self-reported European and African ancestry participants allowed all subjects to select either between receipt of nicotine patch or bupropion (O’Gara et al., 2007).

Ten articles reported results from population-based study designs (i.e., cohort or case-control studies). Of these, 5 studies included data from multiple racial, ethnic, or ancestry groups. Five studies included data exclusively from participants representing White race, European/Caucasian/White ethnicity, or European/EUR ancestry populations.

## Racial, ethnic, and ancestry characteristics across treatments and adverse treatment responses

Almost half of the 79 reviewed studies (49%) investigated the use of two or more treatments simultaneously (e.g., use of a combination of nicotine replacement therapies and bupropion). A similar proportion of these studies were conducted exclusively including White race, White/Caucasian ethnicity, or European ancestry participants (50%) *versus* studies including data from multiple racial, ethnic, or ancestry groups (48%).

**Nicotine Replacement Therapies**. Nicotine replacement therapies (NRT) administer nicotine without the use of a combustible cigarette. NRT help reduce withdrawal symptoms (e.g., cravings) related to nicotine dependence during cigarette abstinence. The most common forms of NRT are the nicotine patch, spray, gum, lozenge, and inhaler. Electronic cigarettes are not an FDA-approved NRT at this time. Further, no genetic association studies have tested use of this product for cessation, and as such, no studies of this product are included in this review. Most pharmacogenetic NRT studies (N = 47) evaluated nicotine patches. Nine studies included data from multiple racial, ethnic, or ancestry groups. There were 37 studies conducted in samples exclusively consisting of White race, White/Caucasian ethnicity, or European ancestry participants. One study exclusively consisted of Black/African American race participants.

The remaining pharmacogenetic NRT studies tested associations between smoking cessation outcomes with nasal spray, nicotine gum, and nicotine lozenges. Ten studies tested nasal spray and all were conducted in White/European samples. Five studies tested nicotine gum. Two of the nicotine gum studies were conducted in Black/African American race participants. Two other studies were conducted in only White race or European ancestry participants. There were six studies of nicotine lozenges. Most of these included lozenges as part of a treatment regimen that included a combination of multiple NRT. One study was conducted in a multigroup sample (White/European ancestry and Black/African American ancestry) and one utilized data from Han Chinese participants in China.

**Non-Nicotine Treatments**. This summary focuses on bupropion and varenicline, the two major classes of non-nicotine treatments for which most pharmacogenetic studies (N = 62) were conducted. These treatments are also most commonly prescribed to support smoking cessation. Eleven studies of other non-nicotine treatments (e.g., naltrexone, venlafaxine, d,1-fenfluramine, nortriptyline) were also conducted.

Bupropion acts by: 1) inhibiting reuptake of dopamine and norepinephrine into the synaptic vesicles, 2) behaving as an allosteric modulator of serotonin receptors (Wilkes, 2008; Zhu et al., 2012), and 3) functioning as a weak nicotine acetylcholine receptor (nAChR) antagonist to reduce the stimulant effects of nicotine on the nicotinic acetylcholine receptors (Wilkes, 2008). Over half of the publications (42 studies, 53%) that were reviewed investigated bupropion.

Eight studies were conducted in multigroup samples. One study combined data from Black and Asian race participants. Of the single group studies, four were conducted in African American race or AFR ancestry samples and 29 were conducted in participants representing White race, European/Caucasian/White ethnicity, or European/EUR ancestry populations. One study was conducted in Korea.

Varenicline acts as a high affinity partial agonist at α6β2-containing nAChRs, which is also important for nicotine dependence (Brunzell, 2012). During lapses of smoking abstinence, it competes with nicotine for nAChR binding sites to reduce nicotine-elicited dopamine release (Faessel et al., 2010). There were 20 papers that investigated varenicline.

Seven studies were conducted in multigroup samples. Of single group studies, one was conducted in an African American race/AFR ancestry sample and 12 studies were conducted in White race, European/Caucasian/White ethnicity, or European/EUR ancestry populations.

**Adverse Treatment Response**. Seventeen studies evaluated adverse side effects to pharmacotherapy in addition to treatment success/abstinence (Chen et al., 2020; Chenoweth et al., 2021; Conti et al., 2008; David et al., 2007a; De Ruyck et al., 2010; King et al., 2012; Lee et al., 2007; Lerman et al., 2002; Lerman et al., 2006; Robinson et al., 2007; Rose et al., 2010; Sarginson et al., 2011; Sun et al., 2012; Swan et al., 2005; Swan et al., 2007; Swan et al., 2012; Zhu et al., 2012) The most commonly studied effects included nausea, cravings, insomnia, mood alterations, and appetite alterations (David et al., 2007a; Rose et al., 2010; King et al., 2012; Sun et al., 2012; Swan et al., 2012; Zhu et al., 2012; Chenoweth et al., 2021). Other studies measured adverse side effects as a composite severity score across multiple adverse events (Sarginson et al., 2011; Chen et al., 2020), the presence of adverse symptoms alongside measures of withdrawal severity (Lerman et al., 2006; Robinson et al., 2007; Conti et al., 2008; De Ruyck et al., 2010), or as a lack of treatment adherence due to the presence of side effects (Swan et al., 2005; Swan et al., 2007). Three studies measured adverse events in multi-group race, ethnicity, or genetic ancestry populations (Rose et al., 2010; Sarginson et al., 2011; Chen et al., 2020). One study was conducted in an African American race sample (Zhu et al., 2012). One study was conducted in China among Han Chinese participants (Sun et al., 2012). Of the papers that evaluated treatment side effects, 13 tested for genetic associations with side effects. Of these, 7 studies detected significant associations (six studies including White race participants exclusively and one multi-race study).

## Discussion

This is the first scoping review that summarizes results on the pharmacogenetics of smoking cessation treatments across racial, ethnic, and genetic ancestry populations. This review identified three major conclusions. First, the majority of articles analyzed data from participants representing White race, European/Caucasian/White ethnicity, or European/EUR ancestry populations and have generally been conducted in the US and United Kingdom. Similarly, most studies *exclusively* consisted of participants representing White race, European/Caucasian/White ethnicity, or European/EUR ancestry populations. Second, four genes were suggested for further investigation with treatment-related smoking abstinence when reviewing studies for common results across studies by race, ethnicity, and ancestry. These have generally been derived from candidate gene studies, with some additional support from recent GWAS results. Third, statistical analyses accounted for race, ethnicity and genetic ancestry using multiple strategies and results summarized below require additional replication. We discuss these results from the perspective of advancing a need to conduct future pharmacogenomic studies of smoking cessation that have increased participant diversity.

The aforementioned results and related conclusions should be evaluated in light of the following limitations. First, we only searched PubMed and as such results may not represent all published pharmacogenomic studies of smoking. However, most if not all, genetically informative research is indexed in PubMed and the likelihood that some articles from other databases were missed is expected to be low. Second, we reported results from any study that included data from non-White participants though their sample sizes may be too small (N < 10) to make strong conclusions. Nevertheless, we considered that a full account of all samples regardless of size be reported in order to produce a comprehensive summary of the current state of pharmacogenetic research incorporating non-White samples. Consequently, we consider common results across studies in aggregate. Third, data from a few studies have been used multiple times for the publication of different genetic associations. Consequently, this review does not account for resampling/re-analysis of datasets across published studies. Nevertheless, it offers a comprehensive perspective by which to consider future directions.

### Genetic variants involved in smoking cessation pharmacotherapy

Of the reviewed articles that included data from participants representing non-White race, non-White ethnicity, or non-European ancestry populations, several significant genetic associations were detected between treatment responses with variants in multiple genes. However, the evidence was strongest for involvement of a gene whose product is involved in nicotine pharmacokinetics (*CYP2A6*), one involved in nicotine and bupropion pharmacokinetics (*CYP2B6*)*,* and two influencing nicotine pharmacodynamics (*CHRNA5 and COMT*). Similar results have also been reported in studies exclusively including White race, European/Caucasian/White ethnicity, or European/EUR ancestry populations.

**FIGURE 4:**
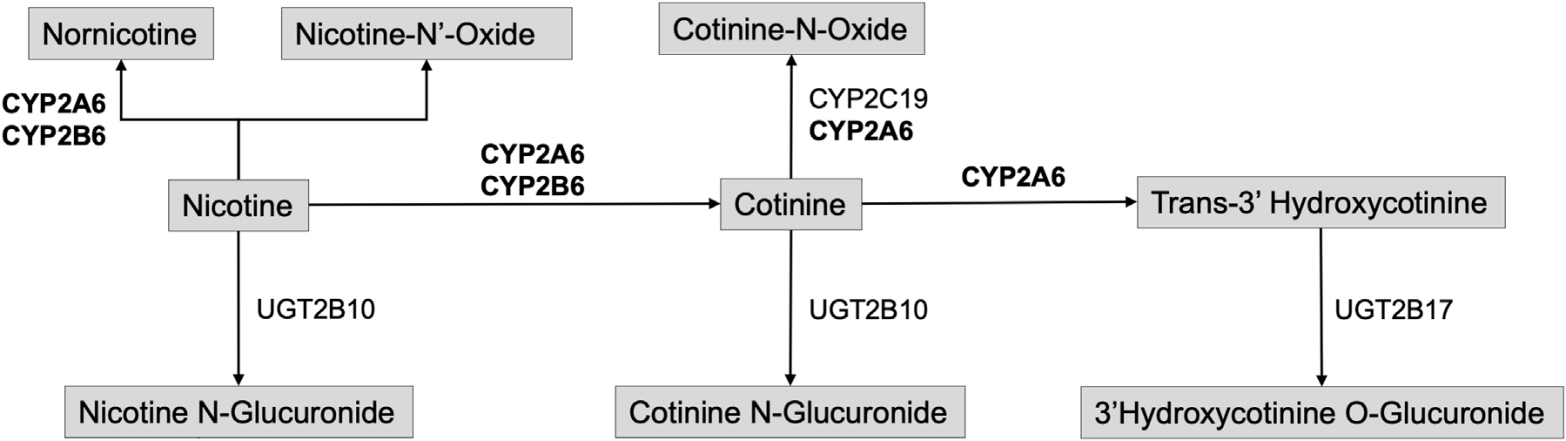
Overview of Major Nicotine Metabolism Pathways. Bolded enzymes highlighted in results.

***CYP2A6***. The metabolic inactivation and clearance of nicotine primarily occurs via the cytochrome P450 pathway through the CYP2A6 enzyme (Figure 4). CYP2A6 metabolizes ∼80% of plasma nicotine into cotinine (COT) through inactivation in the liver. It also further metabolizes COT into 3′hydroxycotinine (3HC). The nicotine metabolite ratio (NMR) is used to measure the rate of nicotine clearance as a ratio of levels of 3′hydroxycotinine *versus* cotinine (3HC/COT) and is considered an established biomarker of nicotine clearance (El-Boraie et al., 2020; El-Boraie et al., 2021).

### Identification of several genetic associations with variants in *CYP2A6* is therefore expected given the biological importance of CYP2A6 in nicotine metabolism

Associations with variants in *CYP2A6* were detected in a multigroup study and two AFR ancestry studies using double-blind randomized placebo-controlled trials as well as one randomized placebo-controlled trial (Ho et al., 2009; Bress et al., 2015; Chenoweth et al., 2018). Additionally, study of nicotine gum in participants indicating African American race detected an association between *CYP2A6* variants and slow and intermediate levels of CYP2A6 metabolism via 3HC/COT measures (Ho et al., 2009). A GWAS reported a significant association between 3HC/COT and multiple variants in *CYP2A6* in a sample of AFR ancestry participants in a study of varenicline and bupropion (Chenoweth et al., 2018). The *CYP2A6* genetic risk score produced from the GWAS results explained a moderate amount of the variance for 3HC/COT (30%–35%) in an AFR ancestry sample. Further, it was significantly associated with nicotine metabolism efficiency (normal vs slow metabolizers via NMR) and demonstrated significant differences in abstinence among normal metabolizers engaged in varenicline use (El-Boraie et al., 2021). Consequently, there is preliminary evidence in samples including non-White race, non-White ethnicity, or non-European ancestry populations focused on *CYP2A6* variants which explains a considerable amount of the variance of nicotine metabolic function and may be useful for future pharmacogenomic applications.

***CYP2B6***. Compared to CYP2A6, the CYP2B6 enzyme represents a minor but clinically significant metabolizer of nicotine (Bloom et al., 2019). Importantly, it is also involved in the hydroxylation of bupropion to hydroxybupropion (Faucette et al., 2000; Hesse et al., 2000; Zanger et al., 2007; Coles & Kharasch, 2008). Approximately 50% of the metabolites resulting from bupropion metabolism is produced in the form of hydroxybupropion. Therefore, CYP2B6 is expected to be important in the pharmacologic activity of bupropion.

A study of AFR participants receiving bupropion were genotyped for CYP2B6*6 and CYP2B6*18. These genotypes were then used to categorize participants as normal-, intermediate-, and slow-metabolizers. The study reported an association between self-reported smoking abstinence at week 7 CYP2B6 metabolizer activity to be mediated by a higher hydroxybuprpion concentration. Consequently, any association between *CYP2B6* genotype and bupropion-aided abstinence occurred through the metabolism of the drug (Chenoweth et al., 2018). Results from a trial of bupropion in an African American sample reported a significant association between higher concentrations of hydroxybupropion and successful abstinence at weeks 3, 7, and 26. Further, the variation of hydroxybupropion concentrations were due to *CYP2B6* variants (Zhu et al., 2012). One study of CYP2B6*4 (rs2279343) reported higher rates of abstinence in a trial of bupropion plus either varenicline or nicotine patch from multiple race groups in Brazil (Tomaz et al., 2015). Another study of varenicline in multiple race groups in Brazil reported a significant association of the rs8109525 variant in *CYP2B6* (Tomaz et al., 2019). Additionally, nausea due to bupropion treatment was associated with a variant (1459C>T) in CYP2B6 in a sample of Caucasians of European ancestry (Lerman et al., 2002). Therefore, *CYP2B6* variants are expected to influence treatment-related smoking abstinence through the metabolic function of CYP2B6 which affects the speed at which bupropion is metabolized. This may also influence the presence of adverse side effects for those receiving bupropion treatment. Given that genetic association results with varenicline were also reported, it is possible that *CYP2B6* variants may also influence the metabolism of this treatment as well.

***CHRNA5***. *CHRNA5* is responsible for production of the α5 nicotinic acetylcholine receptor subunit. The α5 subunit co-assembles with the α3 and β4 subunits and this α5-α3-β4 class of receptors modifies downstream neurotransmitter release when it binds with nicotine (Scholze & Huck, 2020). Associations between treatment-related abstinence and *CHRNA5*, particularly with the rs16969968 variant, were reported in participants representing multiple racial groups across two US and one Brazilian study (Sarginson et al., 2011; Tomaz et al., 2018; Chen et al., 2020). This variant is also one of the most frequently identified in candidate gene association studies and GWAS of smoking phenotypes as well as smoking cessation (Lassi et al., 2016; Erzurumluoglu et al., 2020). Additionally, treatment-induced nausea due to varenicline was associated with variants in *CHRNA5* in a sample of EUR ancestry participants (King et al., 2012).

***COMT***. *COMT* produces catechol-O-methyltransferase, an enzyme which metabolizes dopamine, epinephrine, and norepinephrine in the neuron. This variant has been associated with altering COMT enzymatic activity and in turn affecting dopamine signaling in the prefrontal cortex (Chen et al., 2004; Shield et al., 2004). Two studies of the *COMT* Val158Met variant (rs4680) in studies conducted in Korea and China reported associations with smoking abstinence (Han et al., 2008; Sun et al., 2012). Another US study reported significant associations with two *COMT* variants, rs737865 and rs165599 (Berrettini et al., 2007). A significant association with variants within this gene was also reported in one single race study of European ancestry participants (Johnstone et al., 2007). Taken together, these studies suggest the role of variants whose products regulate downstream neurotransmitter release and metabolism after exposure to nicotine whose genetic effects may be detected in non-EUR ancestry populations.

Most articles reported results from candidate gene studies. Results in this review therefore draw heavily from a body of clinical trials conducting pharmacogenetics research with a focus on a specific set of genes, rather than the pharmacogenomics (across all genes in the genome) studies. An initial focus on candidate gene studies is reasonable given the well-characterized process of nicotine metabolism as well as the substantial costs to conduct clinical utility studies. Some of the candidate gene results have also been reported in GWAS studies. The combination of candidate gene studies and GWAS results are being further expanded towards the development of candidate gene scores across several loci. This approach has been implemented with some success for the assessment of breast cancer risk in multiple ancestry groups (Gao et al., 2022; Ho et al., 2022; Mavaddat et al., 2019). Given positive preliminary evidence for the use of a candidate gene score for *CYP2A6*, this approach has promise for use in treatment of nicotine dependence. Additionally, a total of 13 studies evaluated genetic associations with adverse side effects and only two included data from non-EUR ancestry participants, encouraging additional study in this area since the presence of side effects is an important factor influencing treatment adherence.

### Future research directions for pharmacogenetic studies of smoking cessation

**Accounting for Race/Ethnicity and Genetic Ancestry**. Results from this scoping review renews the call for more diverse samples in pharmacogenetic and pharmacogenomic trials of smoking cessation across racial, ethnic, and groups (Chen et al., 2018b; Saccone et al., 2018; Salloum et al., 2018). Approximately one-quarter (26%) of reviewed articles included data from participants across racial, ethnic, and ancestral groups beyond those defined as White race, White/Caucasian ethnicity, and European/EUR ancestry. This trend is consistent with clinical trial (Lolic et al., 2021) and genetic association research (Sirugo et al., 2019).

***Identification of Race, Ethnicity, Genetic Ancestry, and Country of Study***. Of the 25 studies that included participants representing non-White race, non-White ethnicity, or non-European ancestry populations, 17 used measures of self-reported race or ethnicity and 7 estimated genetic ancestry. There was a wider range of definitions used to measure self-report race, ethnicity, and ancestry in studies that included White race, European/Caucasian/White ethnicity, or European/EUR ancestry populations. There was a high degree of inconsistency in measurement details for self-reported race, ethnicity, and ancestry. This likely reflects a larger issue of ambiguity among researchers related to the conceptualization, discussion, and use of the terms race and ethnicity as well as ancestry (Dauda et al., 2023).

As more pharmacogenetic studies of smoking cessation are conducted, some concerns with respect to the identification of race, ethnicity, or ancestry should be considered. There is a growing need to include genetic ancestry alongside race and ethnicity because 1) genetic ancestry is needed for sample inclusion into large scale consortia with the power to detect significant genetic associations particularly with GWAS data (Chen et al., 2018b) and 2) seemingly homogeneous groups may have population substructure that can be captured by genetic ancestry (Elhaik et al., 2013; Benn Torres et al., 2019) which may result in biased estimates of genetic association due to population stratification (Marchini et al., 2004). Nevertheless, the use and inclusion of genetic ancestry for conducting genetic association studies for diverse samples should be conducted alongside the collection of race and ethnicity data as well as other variables that capture the underlying societal factors influencing smoking cessation. Further, the influence of these variables will vary by country of study. This review identified two cross-country studies (US and Canada) and more are expected in the future as has been the trend in studies of smoking behaviors (Liu et al., 2019). In anticipation of this area of growth, methods that consider inclusion of these sources of variance may be required to reduce heterogeneity across studies.

***Epidemiological Study Design.*** Over half (56%) of the 25 studies that included participants representing non-White race, non-White ethnicity, or non-European ancestry populations (N = 14) conducted double-blind randomized placebo-controlled trials, which are considered to be a gold standard of epidemiological studies. In comparison, 28% of studies conducted exclusively in White race, White ethnicity, or European ancestry samples used this approach. As future studies advance, continued attention and care to such study designs are warranted to ensure results could be readily translated into clinical settings.

***Statistical Analysis***. There were three general classes of analytic strategies used across the 18 multigroup studies: 1) combining samples and conducting analyses across groups without reporting adjustment for race or ethnicity (Robinson et al., 2007; Zhu et al., 2014a; Santos et al., 2020); 2) combining samples across groups and conducting analyses while adjusting for the variance due to race, ethnicity, or genetic ancestry principal components (Cinciripini et al., 2004; O’Gara et al., 2007; Rose et al., 2010; Sarginson et al., 2011; Rocha Santos et al., 2015; Tomaz et al., 2015; Tomaz et al., 2018; Tomaz et al., 2019; Roche et al., 2019) and 3) conducting stratified analyses by racial, ethnic, or genetic ancestry groups (Berrettini et al., 2007; Bress et al., 2015; Chen et al., 2020; El-Boraie et al., 2021; Ton et al., 2007). Thoughtful consideration of the incorporation of genetic ancestry is particularly important to account for population admixture in populations resulting from ancestors consisting of two or more populations such as Latino/Amerindian participants. Strategies for analyzing samples with multiple ancestry groups for use with GWAS data have been detailed elsewhere (Peterson et al., 2019). Briefly, this approach currently includes either conducting analyses separately by ancestry group and later meta-analyzing all groups together or including and adjusting for the variance due genetic ancestry estimates through principal components in tests of association.

Candidate gene association studies rely on *a priori* knowledge regarding biological function, which may yet be incomplete. Further, candidate gene study results can be difficult to replicate, particularly for studies of gene-environment interaction (Duncan & Keller, 2011). This limitation could extend to studies of gene-drug interaction. Consequently, candidate gene association studies have become less popular, and GWAS data and related methods have been encouraged for pharmacogenetic trials of smoking cessation (Chen et al., 2018b). The remainder of this discussion will consider practical issues in the process of developing and conducting GWAS for advancing pharmacogenomic smoking cessation studies. Reviews have already addressed the methodological considerations related to this type of research (e.g., data types, study design, analytic methods) (Chen et al., 2018b; Peterson et al., 2019) and will not be detailed here. However, such analyses cannot be conducted unless the data from diverse populations are collected and available. Practical issues in the collection of such data are needed. In response, we address fundamental considerations within the intersection of clinical applications and utility of the pharmacogenetics research of smoking cessation. We also consider the practical concerns underlying research participation in pharmacogenomic studies within marginalized populations by race and ethnicity that are a high priority target for inclusion in GWAS.

**Unique Challenges in Pharmacogenomic Studies of Smoking Cessation**. Below, we consider the challenges and potential strategies to developing pharmacogenomic studies of smoking cessation with diverse samples since there a need to increase diverse study participation across racial, ethnic and genetic ancestry groups. There are several underlying sources of these concerns, reflecting participant ascertainment strategies, community attitudes towards research, institutional policies in research and medicine, and participant engagement strategies. We consider each and identify strategies to minimize their influence in order to improve participation of marginalized communities.

***Participant Ascertainment***. The articles reviewed reported ascertainment strategies that recruited participants from clinical settings, utilizing digital resources to connect people interested research participation with projects (e.g., research registry platforms), or using community-facing advertising (e.g., advertisements via radio, television, and newspapers, informative presentations at health fairs and libraries, local businesses and religious organizations, and website communication). To date, the steps involved in this stage are rarely detailed. Few of the reviewed publications included information regarding consultation from members of the target populations of a study. This is a missed opportunity because these individuals understand the motivations for research participation. Such one-sided strategies may result in increased time and money spent for data collection and retention. One approach to address this issue is to include a community advisory board (CAB, also known as a community research board or participant advisory board) as part of a research team. A CAB consists of stakeholders outside of academia who do not have formal training in research but have a general interest in the conduct of research and received training in research ethics and review of research. CAB review study details and offer feedback on proposed approaches with a goal of increasing study accessibility to the lay public (Mitchell et al., 2020). Use of these strategies along with others can help to tailor ascertainment strategies, increase participation, and improve retention (Wong et al., 2021; Pancras et al., 2022). A few of the reviewed trials reported use of a CAB in study development (Ahluwalia et al., 2006; Cox et al., 2012; Lerman et al., 2015). In addition to the perspectives of the lead clinicians at a study site, the inclusion of insight from the clinical staff who might be likely to be part of the recruitment process in the trial as part of the study team is also expected to positively impact recruitment (Mentz et al., 2016). Clinical stakeholders also have a unique understanding of the benefits and challenges related to research involvement.

***Institutional Policies That Limit Perceived Benefits and Influence Participation.*** Most articles in this review jointly evaluated multiple pharmacotherapies. Of all treatments, nicotine patches were studied most often (N = 46). Fewer studies assessed bupropion (N = 42) and varenicline (N = 20) although these treatments have greater efficacy (Nollen et al., 2021). This difference in treatment representation, particularly for varenicline, may be due to other factors that could also influence research participation. Institutional policies in the access to treatment is likely to influence public and clinician attitudes and may influence study participation. For example, cost-containment policies in the US that strongly encourage use of generic drugs might have inadvertently prevented Medicaid-covered smokers from obtaining pharmacotherapy, such as varenicline. Varenicline is the most effective smoking cessation medication (Krist et al., 2021). However, there were sustained supply chain limitations in the production of varenicline over several years (US Food and Drug Administration, 2022b). Consequently, between 2010–2015, generic nicotine (including nicotine patches) was most frequently prescribed for smoking cessation to Medicaid beneficiaries, limiting the access and benefit of all pharmacotherapies to promote abstinence.

Similar to the current landscape of treatment, the current benefit realized by the average patient as a result of genetic or pharmacogenetic testing is low and limits the ability of the public to appreciate this tool to improve health. In 2010, the American Affordable Care Act (ACA) established Medicaid as the foundation of the insurance continuum. Medicaid is now the largest or second largest insurer in each state (Bachrach et al., 2015). Medicaid, like all insurers, has a responsibility to ensure that its coverage decisions apply equitably to all of its members. However, a lack of currently relevant cost-effectiveness research in the pharmacogenomics of smoking cessation significantly limits its ability to be sufficiently evaluated within a value-based purchasing context. Although pharmacogenomic testing coverage has been actively considered by Medicaid (Bachrach et al., 2015), few studies have evaluated the cost-effectiveness of pharmacogenetic testing to tailor smoking cessation treatment. To date, one study reported that use of a tailored approach to choosing between nicotine patch and bupropion could be cost-effective under specific conditions (Heitjan et al., 2008a). Such evaluation has not been conducted for varenicline. Consequently, the information typically required to expand access for pharmacogenomic testing to address smoking cessation is not currently available. Therefore, public enthusiasm towards research participation in the pharmacogenomics of smoking cessation is likely to be limited.

### Imagining a more diverse future of pharmacogenomics research for smoking cessation requires continued appreciation for the current landscape

The future that is currently envisioned for the pharmacogenomics of smoking cessation includes utilization of polygenic scores which captures a substantial proportion of the genetic variance that predicts smoking abstinence or biomarker (e.g., 3HC/COT, DNA methylation) levels related to nicotine/treatment metabolism or treatment side effects (Saccone et al., 2018; Ramsey et al., 2021). This future will likely include the creation of polygenic scores that account for diverse allele distributions across populations and captures the magnitude of pharmacogenomic for smoking cessation across the key pharmacologic treatment options (varenicline, bupropion, and NRT). In an effort to facilitate a vision of including pharmacogenomic testing in smoking cessation treatment, researchers in collaboration with other stakeholders will need to thoughtfully consider four current realities. First, it likely that the populations for which researchers are seeking participation in pharmacogenomic clinical trial do not experience a benefit from the treatments offered at the time of study invitation. Consequently, potential participants may experience limited trust towards the clinicians that prescribe such treatments which could extend to smoking cessation researchers. For example, a recent randomized clinical trial of US smokers receiving varenicline, bupropion, and nicotine patch reported greater efficacy in all treatments compared to placebo for White participants. However, varenicline was the only pharmacotherapy with greater efficacy in Black participants (Nollen et al., 2021). Therefore, asking individuals to trust researchers affiliated with a health system would involve some risk-taking from potential participants who are already experiencing vulnerability and limited benefit from the medical system meant to support them.

A reconsideration of the strategies that maximize participant benefit in pharmacogenomic studies of smoking cessation beyond monetary compensation is needed. In the articles that included participants representing non-White race, non-White ethnicity, or non-European ancestry populations, 12 included behavioral counseling or health education to all participants. Six studies did not offer additional support to all participants. In instances where a pharmacotherapy was evaluated, availability of behavioral counseling related to smoking cessation or mental wellness broadly may be considered a welcome benefit of participation. An additional benefit studies may consider includes reducing financial, time, and social barriers through connection to supports beyond smoking cessation (e.g., transportation, wellness, housing). This has also been reported to be an important factor in participation and retention because it offers support in areas of individual need (Wong et al., 2021). Additionally, improving participant comprehension of trial objectives and procedures is needed and will likely require multiple conversations and reminders throughout the duration of the study. This could include return of non-controversial results such as genetic ancestry classifications and related education as exemplified by Genes for Good (Brieger et al., 2019). These actions are likely to establish rapport between a research team and a community of individuals who may be interested in participating as well as in a population of participants. Consequently, when studies emphasize building legitimacy or trust between research teams and participants, they should be able to expect improved recruitment and retention (Wong et al., 2021).

Second, researchers who plan to engage in community-facing recruitment will need to do so while carefully investing in partnerships with communities and the organizations that serve them. In the case of smoking cessation, this may include collaboration with local health departments, non-profit agencies supporting community wellness, independent community pharmacies, local businesses, and the faith-based community who often offer health education and screening related to smoking cessation. The benefits of such partnership may not naturally present immediately, and may require academic teams to support other partner needs while requesting support for research projects. This will include addressing key principles and including community stakeholders as research partners as early as possible in the research process, to include issues such as equal partnership, capacity building, data sharing, community engagement, results dissemination, and community-wide benefits (Parker and Kwiatkowski, 2016; Claw et al., 2018). Community-academic collaborations such as H3Africa offer an example (Dauda & Joffe, 2018). Further, projects that use community-based participatory research principles offer additional strategies (Mitchell et al., 2020).

Third, research teams should consider supporting clinician efficacy to process and act on current evidence from pharmacogenetics test results and their clinical implications. Such effort will support the investment and broader career development of this important group of research stakeholders (Roberts et al., 2019). One concern in advancing pharmacogenomics research is that health providers face challenges in their own genetic literacy when relaying test information to patients (Shuldiner et al., 2013; Hippman & Nislow, 2019; Haidar et al., 2022). This unfamiliarity creates limitations in patient health literacy to engage in informed decision making for medications. In the application of dispensing smoking cessation drugs, clinicians must determine if genetic testing is appropriate, assess the cost-effectiveness considering patient socioeconomic status, and make recommendations based on results (Hippman & Nislow, 2019; Haidar et al., 2022). The overall uncertainty creates barriers to implementing pharmacogenomics in clinical practices. Adding pharmacogenomics results can therefore be perceived as a burden rather than a benefit.

Strategies by which research teams can immediately support clinicians as well as their clinical staff and establish partnership with this group of stakeholders are needed. These include production—perhaps in consultation with these stakeholders—of educational tools (e.g., infographics) that summarize important research results, clinical interpretations related to specific tests, or strategies by which to communicate genetic testing and results in clinician-identified priority areas. Such results-based knowledge could be sent on a regular basis to address some of the aforementioned challenges. This would help to establish trust and rapport with clinicians prior to study development and throughout the lifespan of a project.

Fourth, progress in reducing health disparities requires an acknowledgment of the inherent biases entrenched in systems of biomedical research as well as medical care and decisively change our research patterns to address both areas. This transition is challenging for pharmacogenomics research given its bias towards rapid knowledge generation in response to researcher-related concerns (e.g., grant submission deadlines) which do not readily align with the calendars and priorities of stakeholders who typically support diverse populations. This has historically resulted in the use of quick data collection strategies that favor participation from White race and highly educated populations. It also limits the researcher’s ability to productively partner with stakeholders.

We demonstrated a continued reliance on the use of race and ethnicity as a proxy for genetic ancestry in research, which could unintentionally be translated inaccurately into future clinical application of pharmacological treatment of smoking cessation. Further complicating the systems-level causes of bias in pharmacogenomics research is that disease prevalence and health disparities are predominantly reported by racial and ethnic groups (Goodman & Brett, 2021). This bias is perpetuated by the FDA which frequently utilizes race-based recommendations for genetic testing (US Food and Drug Administration, 2016; Chang et al., 2020; US Food and Drug Administration, 2022a). However, race and ethnicity remain poorly defined variables which are applied across genetic, biologic, environmental and social aspects of health. Moving forward, we suggest embracing and clearly discussing race and ethnicity as complicated social constructs that are critical to understanding smoking cessation. It is well established that the largest drivers of individual health are social factors. It is critical to avoid a reductionist approach to pharmacogenomics that separates genetics and systems level causes of disparities (Parker & Satkoske, 2012). This includes the incorporation of pharmacogenomic and social environmental characteristics of the patients, the latter of which contains race and ethnicity. In the absence of more precise measures of social and cultural factors, these measures remain useful for study inclusion with genetic ancestry (Borrell et al., 2021).

It is also necessary to disentangle discussion of race and ethnicity from genetic and biologic interpretation within study designs and broader applications of research results. This requires more transparency and clear discussion regarding the definitions and uses of race, ethnicity, and genetic ancestry. Such clarification allows for greater detailing of the processes and factors underlying smoking and cessation. This shift will require significant time and investment given the pervasive use of race and ethnicity as a discreet term across societal data (i.e., birth registries, tax data, electronic health records, *etc.*). Additionally, it will be necessary to appropriately incorporate race and ethnicity as social environment variables into genetically-informed models alongside genetic ancestry and other pharmacogenetic variants of interest.

Pharmacogenomic testing for smoking cessation has the potential to address some of the current challenges for effectively maintaining smoking abstinence in all populations (Ramsey et al., 2021). Consequently, the future for the pharmacogenomics of smoking cessation is promising. Realizing that future requires unique investments from the research and clinical communities as well as the agencies that fund these efforts to mend the trust and systematic bias that has influenced genomics research and healthcare (Magavern et al., 2022). Such effort has the potential to generate important discoveries in ways that also prioritize partnership between the academic community and the populations it serves which in turn advances the need to expand the diversity of pharmacogenomic research in smoking cessation.
