# Supplementary material for: A scoping review of smoking cessation pharmacogenetic studies to advance future research across racial, ethnic, and ancestral populations
Source: Front Genet. 2023 Jun 8;14:1103966. doi: 10.3389/fgene.2023.1103966 (PMC10285878; doi:10.3389/fgene.2023.1103966)
Supplement: Supplementary file 1 [file DataSheet1.pdf]

Supplementary Text 1: Variables for Extraction from Identified Studies and Relevant Descriptions

1. **Title:** Article Title
2. **Author:** Last name of the first author of the article
3. **Year:** Year published in journal
4. **Populations Included:** Report of sample size by race/ethnicity.
5. **Determination of race/ethnicity/genetic ancestry:** Report on method used to categorize participants by race/ethnicity or genetic ancestry.
6. **How did they report race/ethnicity/genetic ancestry:** What actual words were used by the authors describe the concepts for race/ethnicity/genetic ancestry
7. **How were race/ethnicity included in statistical analysis- (Non-White or White only, stratified by race, pooled analysis adjusted for race, or pooled analysis not adjusted for race):** Report of how authors handled race in statistical analysis.
8. **Epidemiological study type:** Epidemiology study design used (e.g., Double-Blind Randomized Placebo-Controlled Trial, Cohort Study, etc.) including information about participant ascertainment
9. **Country of Study:** What city/cities and country/countries were the participants recruited from
10. **Psychological or behavioral treatments tested:** Report of any psychological or behavioral counseling included during treatment arms.
11. **Number of treatment arms:** Report of the number of non-placebo pharmacological treatment arms
12. **Duration of treatment arms:** Report of the length of each treatment arm, including information about the timing of controlled quit days.
13. **Drug dose of treatment arms**: Report of the drug, drug dose amount, and including information about any changes throughout treatment duration and any additional pharmaceutical treatments.
14. **Comparison group (placebo or another drug):** Report of the comparison group use, including any information about additional pharmaceutical treatments.
15. **How were treatment arms analyzed? (Controlled for treatment in model, stratified analysis by treatment, or pooled all treatment groups):** Report how treatment arms were handled in statistical analyses, including descriptions of any special cases that do not fit the options provided.
16. **Outcome(s):** Report of the method and timing of smoking cessation measurement, report any secondary smoking cessation measurements as well
17. **Did the study evaluate treatment side effects?**
18. **Genetic study design (Candidate gene, GWAS, polygenic risk score, candidate gene risk score):** Report of the type of genetic study design.
19. **Genetic assessment type (genetic association, gene-by-treatment interaction, or both):** Report of the types of genetic assessments used in statistical analyses, including genetic association with smoking cessation only, gene-by-treatment interaction only, or both.
20. **Genes investigated:** Report of the genes investigated.
21. **Reviewer summary of relevant results with regards to race:** Summary of relevant pharmacogenetic smoking cessation results provided by article reviewer.
22. **Are there comparable studies in European/White-only participant articles? (Yes/No, if yes provide a reference):** Report of identified European/White-only articles using similar source populations, interrogating similar genes, or other similar study designed, reported at reviewer discretion.

Supplementary Table 1. Summary of Pharmacogenomic Smoking Cessation Studies by Genetic Epidemiological Study Design and Study Participants Across Racial Groups, Ethnic Groups, Genetic Ancestry Groups, and Study Countries*

Genetic Study Design	Asian Race (USA/UK/Brazil), China, Korea	AFR Ancestry (US), Black Race (USA/UK/Brazil), African American Race (US)	Hispanic Race (USA), Hispanic Ethnicity (USA), Native American Race (USA), Other Race (USA), Non-White Race (Brazil), Amerindian Race (Brazil)	EUR Ancestry (USA/Canada), White Race (USA/UK/Brazil), European Ethnicity (USA), European Self-Report Ancestry (UK), Netherlands
Candidate Gene	Ton et al. (2007), Han et al. (2008), Tomaz et al. (2018), Sarginson et al. (2011), Sun et al. (2012), Zhu et al. (2014b)	Cinciripini et al. (2004), Zhu et al. (2012), Zhu et al. (2014a), Zhu et al. (2014b), Chenoweth et al. (2022), O'Gara et al. (2007), Ton et al. (2007), Berretitini et al. (2007), Ho et al. (2009), Roche et al. (2019), Tomaz et al. (2018), Sarginson et al. (2011), Chen et al. (2020)	Cinciripini et al. (2004), Ton et al. (2007), Tomaz et al. (2015), Tomaz et al. (2018), Tomaz et al. (2019), Roche et al. (2019), Sarginson et al. (2011), Santos et al. (2015), Munafo et al. (2013), Robinson et al. (2006)	Ray et al. (2007), Lee et al. (2012), Lee et al. (2007), David et al. (2013), Conti et al. (2008), Swan et al. (2012), Hutchinson et al. (2007), Zhu et al. (2014b), Ruyck et al. (2010), Bergen et al. (2014), Bergen et al. (2015), David et al. (2008a), Hu et al. (2006), Verde et al. (2014), Lerman et al. (2002), Quintana et al. (2013), Heitjan et al. (2008), O'Gara et al. (2007), David et al. (2007a), Lerman et al. (2006), Ton et al. (2007), Lerman et al. (2003), Swan et al. (2007), Berretitini et al. (2007), Lerman et al. (2015), Robinson et al. (2006), Santos et al. (2015), Swan et al. (2005), Tydale et al. (2015), Roche et al. (2019), Tomaz et al. (2015), Tomaz et al. (2018), Tomaz et al. (2019), Ware et al. (2015), King et al. (2012), Gold et al. (2012), Johnstone et al. (2004), Johnestone et al. (2007), Guo et al. (2010), Sarginson et al. (2011), Glatard et al. (2017), Chen et al. (2012), Chen et al. (2014), Chen et al. (2015), Chen et al. (2020), Leventhal et al. (2012), Lerman et al. (2004), David et al. (2008b), Bergen et al. (2013a), Bergen et al. (2013b), Munafo et al. (2007), Munafo et al. (2009), Munafo et al. (2013), David et al. (2007b), Breitling et al. (2010), Ashare et al. (2013), Cameli et al. (2018), Cinciripini et al. (2004), Dahl et al. (2016), Pintarelli et al. (2017), Quaak et al. (2011), Turner et al. (2014)
GWAS	-	Chenoweth et al. (2018)	-	Chenoweth et al. (2021), Uhl et al. (2010a), Uhl et al. (2010b), Uhl et al. (2008)
Gene Expression	-	-	Santos et al. (2020)	Santos et al. (2020)
Genome-wide Polygenic Score	-	Rose et al. (2010)	Rose et al. (2010)	Rose et al. (2010)
Candidate gene risk score	-	Bress et al. (2015), El-Boraie et al. (2021)	-	Bress et al. (2015), Chen et al. (2018), El-Boraie et al. (2021), El-Boraie et al. (2020)

*Studies that did not report a measure of race, ethnicity, or ancestry are included by country of study

Supplementary Table 2. Summary of Pharmacogenomic Smoking Cessation Studies by Epidemiological Study Design and Study Participants Across Racial Groups, Ethnic Groups, Genetic Ancestry Groups, and Study Countries*

Epidemiological Study Design	Asian Race (USA/UK/Brazil), China, Korea	AFR Ancestry (US), Black Race (USA/UK/Brazil), African American Race (US)	Hispanic Race (USA), Hispanic Ethnicity (USA), Native American Race (USA), Other Race (USA), Non-White Race (Brazil), Amerindian Race (Brazil)	EUR Ancestry (USA/Canada), White Race (USA/UK/Brazil), European Ethnicity (USA), European Self-Report Ancestry (UK), Netherlands
Double-Blind Randomized Placebo-Controlled Trial	Ton et al. (2007), Sarginson et al. (2011), Sun et al. (2012), Zhu et al. (2014b)	Zhu et al. (2012), Zhu et al. (2014a), Zhu et al. (2014b), Ton et al. (2007), Berretitini et al. (2007), Bress et al. (2015), Ho et al. (2009), Rose et al. (2010), Roche et al. (2019), Sarginson et al. (2011), Chen et al. (2020), Cinciripini et al. (2004)	Ton et al. (2007), Sarginson et al. (2011), Roche et al. (2019), Rose et al. (2010), Robinson et al. (2006), Cinciripini et al. (2004)	Uhl et al. (2008), David et al. (2013), Conti et al. (2008), Lee et al. (2012), Zhu et al. (2014b), David et al. (2007a), Lerman et al. (2006), Ton et al. (2007), Berretitini et al. (2007), Bress et al. (2015), Lerman et al. (2010), Robinson et al. (2006), Hu et al. (2006), Rose et al. (2010), Roche et al. (2019), King et al. (2012), Sarginson et al. (2011), Chen et al. (2020), Leventhal et al. (2012), David et al. (2008b), Bergen et al. (2013a), Bergen et al. (2013b), Munafo et al. (2007), David et al. (2007b), Cinciripini et al. (2004)
Randomized Placebo-Controlled Trial	-	Chenoweth et al. (2018), El-Boraie et al. (2021), Chenoweth et al. (2022)	Munafo et al. (2013)	Chenoweth et al. (2021), Uhl et al. (2010a), Chen et al. (2018), Lee et al. (2007), El-Boraie et al. (2021), Ruyck et al. (2010), Bergen et al. (2014), Bergan et al. (2015), David et al. (2008a), Lerman et al. (2002), Quintana et al. (2013), Heitjan et al. (2008), Uhl et al. (2010b), El-Boraie et al. (2020), Lerman et al. (2003), Tyndale et al. (2015), Ware et al. (2015), Chen et al. (2012), Chen et al. (2014), Chen et al. (2015), Munafo et al. (2009), Munafo et al. (2013), Ashare et al. (2013), Quaak et al. (2011)
Open Label Randomized Trial	Sarginson et al. (2011)	Sarginson et al. (2011)	Sarginson et al. (2011)	Ray et al. (2007), Lee et al. (2012), Hutchinson et al. (2012), Swan et al. (2012), Verde et al. (2014), Swan et al. (2007), Gold et al. (2012), Swan et al. (2005), Sarginson et al. (2011), Lerman et al. (2004), Dahl et al. (2006), Turner et al. (2014)
Other Clinical Trial	Han et al. (2008)	O'Gara et al. (2007)	-	O'Gara et al. (2007)
Population Based	Tomaz et al. (2018)	Tomaz et al. (2018)	Santos et al. (2015), Santos et al. (2020), Tomaz et al. (2015), Tomaz et al. (2018), Tomaz et al. (2019)	Santos et al. (2015), Santos et al. (2020), Tomaz et al. (2015), Tomaz et al. (2018), Tomaz et al. (2019), Glatard et al. (2017), Chen et al. (2012), Breitling et al. (2010), Cameli et al. (2018), Pintarelli et al. (2017)

*Studies that did not report a measure of race, ethnicity, or ancestry are included by country of study

Supplementary Table 3. Summary of Pharmacogenomic Smoking Cessation Studies by Treatment and Study Participants Across Racial Groups, Ethnic Groups, Genetic Ancestry Groups, and Study Countries*

Treatment	Asian Race or Ethnicity (USA/UK/Brazil), Pacific Islander Race or Ethnicity (USA/UK/Brazil), China, Korea	AFR Ancestry (US), Black Race (USA/UK/ Brazil), African American Race (US)	Hispanic Race or Ethnicity (USA), Native American Race (USA), Other Race (USA), Non-White Race (Brazil), Amerindian Race (Brazil)	EUR Ancestry (USA/Canada), White Race (USA/UK/Brazil), European Ethnicity (USA), European Self-Report Ancestry (UK), Netherlands
Nicotine Patch	Tomaz et al. (2018), Sarginson et al. (2011)	Chenoweth et al. (2018), El-Boraie et al. (2021), Bress et al. (2015), Rose et al. (2010), O'Gara et al. (2007), Roche et al. (2019), Tomaz et al. (2018), Sarginson et al. (2011), Chen et al. (2020)	Roche et al. (2019), Santos et al. (2015), Tomaz et al. (2015), Tomaz et al. (2018), Sarginson et al. (2011), Rose et al. (2010), Munafo et al. (2013)	Ray et al. (2007), Chenoweth et al. (2021), Uhl et al. (2010), Lee et al. (2012), Chen et al. (2018), Uhl et al. (2008), El-Boraie et al. (2021), Hutchison et al. (2007), Ruyck et al. (2010), Bergen et al. (2014), David et al. (2008a), Verde et al. (2014), Quitana et al. (2013), Uhl et al. (2010), El-Boraie et al. (2020), O'Gara et al. (2007), Lerman et al. (2006), Bress et al. (2015), Lerman et al. (2014), Rose et al. (2010), Santos et al. (2015), Tyndale et al. (2015), Roche et al. (2019), Tomaz et al. (2015), Tomaz et al. (2018), Ware et al. (2015), Gold et al. (2012), Johnstone et al. (2004), Johnstone et al. (2007), Sarginson et al. (2011), Glatard et al. (2017), Chen et al. (2012), Chen et al. (2014), Chen et al. (2015), Chen et al. (2020), Lerman et al. (2004), David et al. (2008b), Bergen et al. (2013), Munafo et al. (2007), Munafo et al. (2013), David et al. (2007), Breitling et al. (2010), Ashare et al. (2013), Dahl et al. (2006), Pintarelli et al. (2007), Turner et al. (2014)
Nasal Spray	-	-	-	Ray et al. (2007), Lee et al. (2012), Uhl et al. (2008), Hutchison et al. (2007), Quitana et al. (2013), Lerman et al. (2006), Gold et al. (2012), Lerman et al. (2004), Ashare et al. (2013), Dahl et al. (2006)
Nicotine Gum	-	Zhu et al. (2014a), Ho et al. (2009), Chenoweth et al. (2018)	-	El-Boraie et al. (2020), Ware et al. (2015)
Lozenge	Sun et al. (2012)	Chen et al. (2020)	-	Chen et al. (2018), Chen et al. (2012), Chen et al. (2014), Chen et al. (2015), Chen et al. (2020)
Varenicline	Tomaz et al. (2018)	Chenoweth et al. (2018), El-Boraie et al. (2021), Tomaz et al. (2018), Chen et al. (2020)	Santos et al. (2015), Santos (2020), Tomaz et al. (2015), Tomaz et al. (2018), Tomaz et al. (2019)	Chenoweth et al. (2021), El-Boraie et al. (2021), El-Boraie et al. (2020), Swan et al. (2012), Bergen et al (2014), Bergen et al. (2015), Santos et al. (2015), Santos et al. (2020), Tyndale et al. (2015), Tomaz et al. (2015), Tomaz et al. (2018), Tomaz et al. (2019), King et al. (2012), Glatard et al. (2017), Chen et al. (2015), Chen et al. (2020), Bergen et al. (2013), Cameli et al. (2018), Pintarelli et al. (2017)
Bupropion	Zhu et al. (2014b), Han et al. (2008), Tomaz et al. (2018), Sarginson et al. (2011)	Chenoweth et al. (2018), Zhu et al. (2012), Zhu et al. (2014a), O'Gara et al. (2007), El-Boraie et al. (2021), Zhu et al. (2014b), Chenoweth et al. (2022), Berretitini et al. (2007), Tomaz et al. (2018), Sarginson et al. (2011)	Santos et al. (2015), Tomaz et al. (2015), Tomaz et al. (2018), Sarginson et al. (2011)	Lee et al. (2012), Chen et al. (2018), Lee et al. (2007), Uhl et al. (2008), David et al. (2013), El-Boraie et al. (2021), Conti et al. (2008), Zhu et al. (2014b), Bergen et al. (2014), Hu et al. (2006), Verde et al. (2014), Lerman et al. (2002), Quintana et al. (2013), Heitjan et al. (2008), El-Boraie et al. (2020), O'Gara et al. (2007), David et al. (2007), Lerman et al. (2006), Lerman et al. (2003), Swan et al. (2007), Berretitini et al. (2007), Santos et al. (2015), Swan et al. (2005), Tomaz et al. (2015), Tomaz et al. (2018), King et al. (2012), Gold et al. (2012), Guo et al. (2010), Sarginson et al. (2011), Chen et al. (2012), Leventhal et al. (2012), Bergen et al. A (2013), Bergen et al. B (2013), Breitling et al. (2010), Ashare et al. (2013), Quaak et al. (2011), Pintarelli et al. (2017)
Other	Ton et al. (2007), Sun et al. (2012)	Ton et al. (2007), Bress et al. (2015), Cinciripini et al. (2004)	Ton et al. (2007), Robinson et al. (2006), Cinciripini et al. (2004)	Bergen et al. (2015), Ton et al. (2007), Robinson et al. (2006), Chen et al. (2012), Chen et al. (2015), Ashare et al. (2013), Cinciripini et al. (2004), Bress et al. (2015)

*Studies that did not report a measure of race, ethnicity, or ancestry are included by country of study

Supplementary Table 4. Distribution of Studies by Treatment, Genetic Study Design, and Epidemiological Study Designs Across Racial Groups, Ethnic Groups, Genetic Ancestry Groups, and Study Countries*

Category	Asian Race or Ethnicity (USA/UK/Brazil), Pacific Islander Race or Ethnicity (USA/UK/Brazil), China, Korea	AFR Ancestry (US), Black Race (USA/UK/ Brazil), African American Race (US)	Hispanic Race or Ethnicity (USA), Native American Race (USA), Other Race (USA), Non-White Race (Brazil), Amerindian Race (Brazil)	EUR Ancestry (USA/Canada), White Race (USA/UK/Brazil), European Ethnicity (USA), European Self-Report Ancestry (UK), Netherlands
**Genetic Study Design**				
Candidate Gene	6	13	10	62
GWAS	0	1	0	4
Gene Expression	0	0	1	1
Genome-wide Polygenic Score	0	1	1	1
Candidate Gene Risk Score	0	2	0	4
**Epidemiological Study Design**				
Double-Blind Randomized Placebo-Controlled Trial	4	12	6	25
Randomized Placebo-Controlled Trial	0	3	1	24
Open Label Randomized Trial	1	1	1	12
Other Clinical Trial	1	1	0	1
Population Based	1	1	5	10
**Treatment Type**				
Nicotine Patch	2	10	7	46
Nasal Spray	0	0	0	10
Nicotine Gum	0	3	0	2
Lozenge	1	1	0	5
Varenicline	1	4	5	19
Bupropion	4	10	4	37
Other	2	3	3	8
